# Supplementary material for: A Genomic Analysis of the Bacillus Bacteriophage Kirovirus kirovense Kirov and Its Ability to Preserve Milk
Source: Int J Mol Sci. 2023 Aug 9;24(16):12584. doi: 10.3390/ijms241612584 (PMC10454425; doi:10.3390/ijms241612584)
Supplement: Supplementary file 1 [file ijms-24-12584-s001.zip › ijms-2508234-supplementary.pdf]

A genomic analysis of the *Bacillus cereus* bacteriophage *Kirovirus kirovense* Kirov and its ability to preserve milk.

Olesya A. Kazantseva[1,†,*], Anna V. Skorynina[1,†], Emma G. Piligrimova[1], Natalya A. Ryabova[1,2] and Andrey M. Shadrin[1,*]

[1] G.K. Skryabin Institute of Biochemistry and Physiology of Microorganisms, Pushchino Scientific Center for Biological Research of the Russian Academy of Sciences, Federal Research Center, Prospect Nauki, 5, 142290 Pusphchino, Russia

[2] Institute of Protein Research RAS, Institutskaya st., 4, 142290 Pushchino, Russia

* Correspondence: olesyakazantseva@bk.ru (O.A.K.); andrey2010s@gmail.com (A.M.S.).

† These authors contributed equally to this work.

Supplementary Materials:

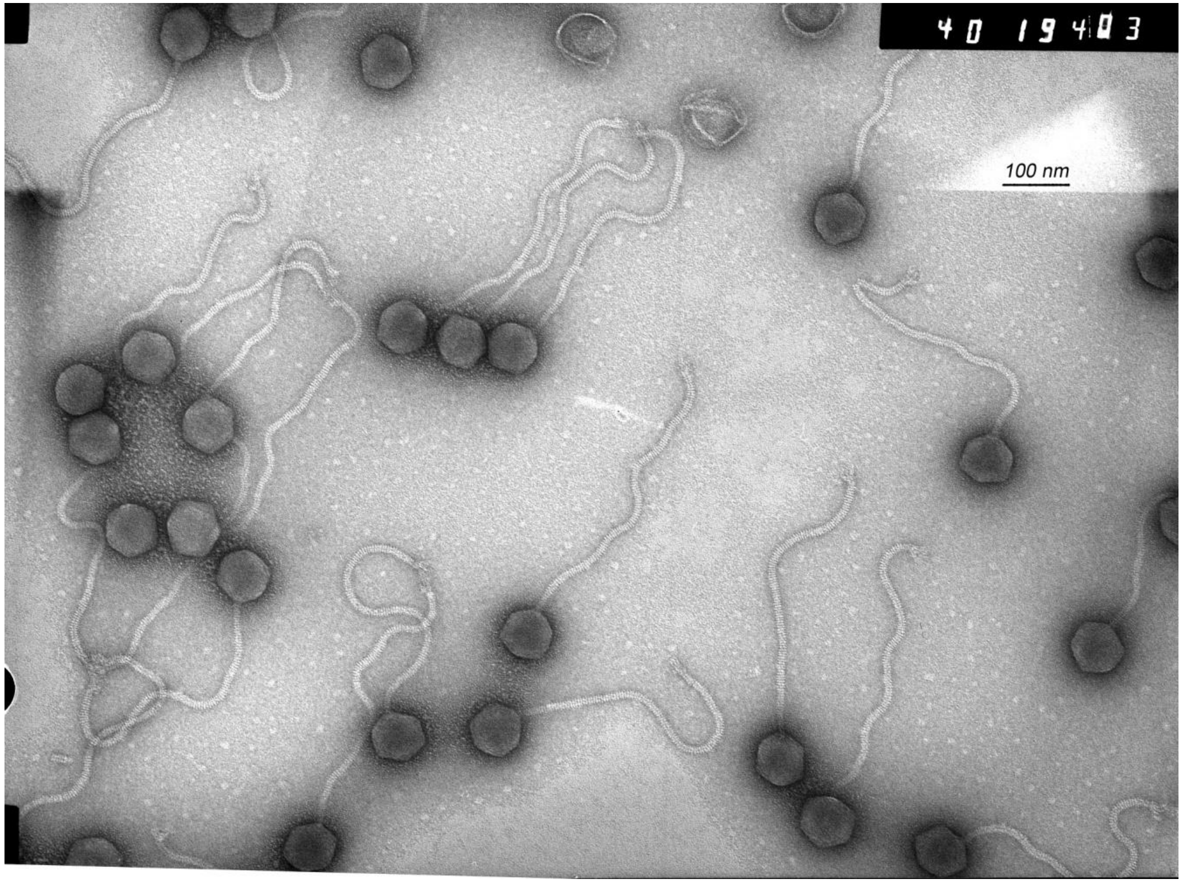

Figure S1. Transmission electron microscopy of *Bacillus* phage *Kirovirus kirovense* Kirov. This is the original TEM micrograph used to generate Figure 1 in the main text. The image was taken using a JEM-100 (JEOL, Japan) transmission electron microscope at 80 kV accelerating voltage on Kodak film SO-163 (Kodak, Cat. # 74144, Hatfield, PA, USA).

Table S1. Primers used to locate the genome ends of *Kirovirus kirovense* Kirov.

**Purpose**	**Primer name**	**Primer sequence (5’-3’)**	№PCR	Template
Determination of the left genome end	kir1_end3_L	TTATGCTGTCTATTGAGAGGATTA	I PCR	genomic DNA of Kirov after DNA tailing reaction
	AncherdT16V	GACCACGCGTATCGATGTCGACTTTTTTTTTTTTTTTTV		
	kir1_end2_L	CTGAGTGGTCGGGTTGTAG	II PCR	I PCR product
	Anch	GACCACGCGTATCGATGTCGAC		
Determination of the right genome end	kir1_end4_R	CCAAATGGTATGAGTTGGGTTA	I PCR	genomic DNA of Kirov after DNA tailing reaction
	AncherdT16V	GACCACGCGTATCGATGTCGACTTTTTTTTTTTTTTTTV		
	kir1_end5_R	TGGTGTTTATTGCGGTGTTTA	II PCR	I PCR product
	Anch	GACCACGCGTATCGATGTCGAC		

Table S2. The host range of *Bacillus* phage *Kirovirus kirovense* Kirov determined on 42 *Bacillus* strains.

No.	Organism	Strain	Source	Phage lysis	No	Organism	Strain	Source	Phage lysis
1	*B. cereus*	VKM B-13	VKM	+	22	*B. cereus*	ATCC 4342	ATCC	+
2	*B. cereus*	VKM B-15	VKM	-	23	*B. cereus*	ATCC 14893	probiotic“Bactis ubtil”[73]	+
3	*B. cereus*	VKM B-370	VKM	+	24	*B. thuringiensis*	VKM B-83	VKM	+
4	*B. cereus*	VKM B-373	VKM	+	25	*B. thuringiensis*	VKM B-84	VKM	+
5	*B. cereus*	VKM B-374	VKM	-	26	*B. thuringiensis*	VKM B-85	VKM	+
6	*B. cereus*	VKM B-383	VKM	+	27	*B. thuringiensis*	VKM B-440	VKM	-
7	*B. cereus*	VKM B-445	VKM	-	28	*B. thuringiensis*	VKM B-443	VKM	-
8	*B. cereus*	VKM B-473	VKM	-	29	*B. thuringiensis*	VKM B-446	VKM	-
9	*B. cereus*	VKM B-491	VKM	+	30	*B. thuringiensis*	VKM B-447	VKM	-
10	*B. cereus*	VKM B-504^{T}	VKM	+	31	*B. thuringiensis*	VKM B-450	VKM	-
11	*B. cereus*	VKM B-681	VKM	-	32	*B. thuringiensis*	VKM B-453	VKM	-
12	*B. cereus*	VKM B-682	VKM	-	33	*B. thuringiensis*	VKM B-454	VKM	-
13	*B. cereus*	VKM B-683	VKM	-	34	*B. thuringiensis*	VKM B-1555	VKM	-
14	*B. cereus*	VKM B-684	VKM	-	35	*B. thuringiensis*	VKM B-1557	VKM	-
15	*B. cereus*	VKM B-686	VKM	-	36	*B. thuringiensis*	ATCC 35646	ATCC	-
16	*B. cereus*	VKM B-687	VKM	-	37	*B. weihenstephanensis*	KBAB4	[74]	-
17	*B. cereus*	VKM B-688	VKM	-	38	*B. megaterium*	MS941	MoBiTec	-
18	*B. cereus*	VKM B-771	VKM	-	39	*B. flexus*		Laboratory collection	-
19	*B. cereus*	VKM B-810	VKM	-	40	*B. subtillis*	168 His III	Laboratory collection	-
20	*B. cereus*	VKM B-811	VKM	-	41	*B. subtillis*	WB 800n	MoBiTec	-
21	*B. cereus*	VKM B-812	VKM	+	42	*B. pumilus*	AVS-01	Laboratory collection	-

Source abbreviations: VKM: All-Russian Collection of Microorganisms; ATCC: American Type Culture Collection; MoBiTec: company MoBiTec GmbH, Germany.

Table S3. Annotation of *Bacillus* phage *Kirovirus kirovense* Kirov.

ORF№	Start codon	Stop codon	Strand	Blast results		Conserved domains, Blast		Hhpred results	Annotation
				Name	E-val	Name, (region)	E-val	(Prob./E-val)	
1	916	602	-	-	-	-	-	('-', '-')	hp
2	1130	933	-	hp	hp	-	-	PF06925.12; MGDG_synth; Monogalactosyldiacylglycerol (MGDG) synthase (62.91/5.70e+00)	hp
3	1503	1117	-	hp	hp	-	-	('-', '-')	hp
4	1781	1503	-	hp	hp	-	-	KOG1597; Transcription initiation factor TFIIB [Transcription](95.61/1.20e-02)<br><br>d1nuia2; g.41.3.2 (A:10-63) Zinc-binding domain of primase-helicase {Bacteriophage T7 [TaxId: 10760]} (95.16/2.60e-02)<br><br>PF11781.9; zf-RRN7; Zinc-finger of RNA-polymerase I-specific TFIIB, Rrn7(94.23/3.70e-02)	Zinc-binding domain containing protein
5	2016	1822	-	hp	hp	-	-	('-', '-')	hp
6	2224	2009	-	hp	hp	-	-	('-', '-')	hp
7	2604	2224	-	Phage protein (N4 Gp49/phage Sf6 gene 66) family [uncultured *Caudovirales* phage]	1.0e-08	Phage_gp49_66 (26-81)	7.09e-14	PF13876.7; Phage_gp49_66; Phage protein (N4 Gp49/phage Sf6 gene 66) family (99.97/1.10e-30)	phage protein (N4 Gp49/phage Sf6 gene 66) family

				Phage protein (N4 Gp49/phage Sf6 gene 66) family [uncultured *Caudovirales* phage]<br><br>Sf6 protein [*Podoviridae* sp. ctda_1]	2.0e-08<br><br>7.0e-08				
8	2842	2597	-	-	-	-	-	d1tq1a_; c.46.1.3 (A:) Thiosulfate sulfurtransferase/Senescence-associated protein {Thale cress (*Arabidopsis thaliana*) [TaxId: 37(71.59/3.3)<br><br>d1repc2 a.4.5.10 (C:144-246) RepE54 {*Escherichia coli*, mini-F plasmid [TaxId: 562]} (75.16/5.4)	hp
9	3165	2893	-	hp	hp	-	-	PF06005.13; ZapB ; Cell division protein ZapB(91.97/2.6)	hp
10	3984	3220	-	hp	hp	-	-	d2ox7a1; b.172.1.1 (A:4-145) Hypothetical protein EF1440 {*Enterococcus faecalis* [TaxId: 1351]} (99.8/7.40e-19)<br><br>d2p84a1; b.172.1.1 (A:4-135) Orf041 product {*Staphylococcus* phage 37 [TaxId: 320840]} (99.77/7.30e-18)<br><br>PF09643.11; YopX; YopX protein (99.68/4.20e-16)	YopX-like protein
11	6404	4110	-	putative viral helicase-like protein [*Bacillus* phage PBC2]	0.0e+00<br><br>0.0e+00	recD_rel (55-734)<br><br>RecD (111-738)	4.74e-104<br><br>6.76e-77	COG0507; RecD; ATP-dependent exoDNAse (exonuclease V), alpha subunit, helicase superfamily I [Replication, recombination and repa(100.0/9.80e-59)	RecD-like DNA helicase

				exodeoxyribonuclease V alpha chain [*Bacillus* phage vB_BcoS-136]<br><br>RecD-like DNA helicase [*Bacillus* phage Izhevsk]	0.0e+00	DEXSc_RecD-like (359-486)	6.11e-36	PF02689.15; Herpes_Helicase; Helicase (99.96/3.50e-28)<br><br>d1w36d1; c.37.1.19 (D:2-360) Exodeoxyribonuclease V alpha chain (RecD) {*Escherichia coli* [TaxId: 562]}(99.95/7.80e-26)	
12	6636	7472	+	putative StbA plasmid stability protein [*Bacillus* phage PBC4]<br><br>putative StbA plasmid stability protein [*Bacillus* phage pW4]<br><br>actin-like protein [*Sporosarcina* phage Lietuvens]	1.0e-20<br><br>2.0e-17<br><br>9.0e-16	ParM_like (8-269)<br><br>ALP_N (11-149)	4.87e-18<br><br>2.28e-09	cd10227; ParM_like; Plasmid segregation protein ParM and similar proteins. ParM is a plasmid-encoded bacterial homolog of actin,(99.92/1.40e-23)<br><br>PF06406.12; StbA ; StbA protein(99.9/1.50e-21)<br><br>COG1077; MreB; Actin-like ATPase involved in cell morphogenesis [Cell cycle control, cell division, chromosome partitioning].(99.88/6.50e-21)	ParM-like protein
13	7459	7773	+	putative ParG-like protein [*Bacillus* phage Izhevsk]	3.0e-33	-	-	d1x93a1; a.43.1.3 (A:31-73) Uncharacterized protein HP0222 {*Helicobacter pylori* [TaxId: 210]}(95.6/4.30e-02)<br><br>d1baza_ a.43.1.1 (A:) Arc repressor {*Salmonella* bacteriophage P22 [TaxId: 10754]} (92.73/0.56)<br><br>d1p94a_a.43.1.3 (A:) Plasmid partition protein ParG {*Salmonella enterica* [TaxId: 28901]} (91.49/0.39)	putative ParG-like protein
14	7918	8124	+	hp	hp	-	-	('-', '-')	hp

15	8320	8817	+	hp	hp	-	-	KOG4631; NADH:ubiquinone oxidoreductase, NDUFB3/B12 subunit [Energy production and conversion] (85.11/3.50e+00)	hp
16	8827	9522	+	M23 peptidase domain containing protein [*Bacillus* phage PBC2]<br><br>endopeptidase [*Bacillus* phage Izhevsk]<br><br>M23 peptidase domain containing protein [*Bacillus* phage pW2]	5.0e-122<br><br>3.0e-119<br><br>2.0e-114	Peptidase_M23 (26-125)	1.77e-17	COG0739; NlpD; Murein DD-endopeptidase MepM and murein hydrolase activator NlpD, contain LysM domain [Cell wall/membrane/envelope (99.54/3.70e-13)<br><br>d1qwya_; b.84.3.2 (A:) Peptidoglycan hydrolase LytM {*Staphylococcus* aureus [TaxId: 1280]} (99.51/9.40e-13)<br><br>PF01551.23; Peptidase_M23; Peptidase family M23 (98.39/2.70e-5)	M23 peptidase domain containing protein
17	9547	9699	+	hp	hp	-	-	PF05508.12; Ran-binding; RanGTP-binding protein (83.56/1.90e+00)	hp
18	9700	12507	+	p-loop containing nucleoside triphosphate hydrolase [*Bacillus* phage PBC2]<br><br>p-loop containing nucleoside triphosphate hydrolase [*Bacillus* phage pW2]<br><br>AAA-like domain protein [*Bacillus* phage vB_BcoS-136]	0.0e+00<br><br>0.0e+00<br><br>0.0e+00	-	-	PF19044.1; P-loop_TraG; TraG P-loop domain(99.96/1.20e-26)<br><br>d1e9ra_; c.37.1.11 (A:) Bacterial conjugative coupling protein TrwB {*Escherichia coli* [TaxId: 562]}(99.95/2.50e-25)<br><br>COG3451; VirB4; Type IV secretory pathway, VirB4 component [Intracellular trafficking, secretion, and vesicular transport].(99.95/4.70e-25)	ATP binding domain TrwB-like protein
19	12546	13316	+	Replic_Relax superfamily protein [*Bacillus* phage Izhevsk]	1.0e-121	-	-	PF13814.7; Replic_Relax; Replication-relaxation(99.81/7.60e-19)	Replic_Relax superfamily protein

				gp27.3 [*Bacillus* virus SPO1]<br><br>plasmid replication-relaxation protein [*Bacillus* phage CampHawk]	1.0e-14<br><br>1.0e-14			d1ub9a_; a.4.5.28 (A:) Hypothetical protein PH1061 {*Pyrococcus horikoshii* [TaxId: 53953]}(97.42/1.50e-03)<br><br>d1t6sa2; a.4.5.60 (A:86-162) Segregation and condensation protein B, ScpB {*Chlorobium tepidum* [TaxId: 1097]}(97.35/1.60e-03)	
20	13544	14560	+	Xer family site-specific tyrosine recombinase [*Bacillus* phage Izhevsk]<br><br>site-specific tyrosine recombinase [*Bacillus* phage pW2]<br><br>site-specific tyrosine recombinase [*Bacillus* phage vB_BcoS-136]	0.0e+00<br><br>0.0e+00<br><br>2.0e-162	Phage_integrase (130-287)<br><br>recomb_XerD (34-193)<br><br>DNA_BRE_C (145-281)	9.11e-08<br><br>3.07e-06<br><br>4.25e-06	COG4973; XerC; Site-specific recombinase XerC [Replication, recombination and repair].(100.0/8.80e-32)<br><br>COG4974; XerD; Site-specific recombinase XerD [Replication, recombination and repair].(100.0/2.10e-30)<br><br>COG4342; COG4342; Intergrase/Recombinase [Mobilome: prophages, transposons].(99.89/3.20e-22)	Xer family site-specific tyrosine recombinase
21	14642	14893	+	hp	hp	-	-	('-', '-')	hp
22	14969	15205	+	hp	hp	-	-	PF18287.2; ; Hfx_Cass5 ; Integron Cassette Protein Hfx_Cass5(84.07/1.60e+00)	hp
23	16292	15444	-	putative lambda O-type replication initiator [*Bacillus* phage Izhevsk]<br><br>putative transcriptional regulator [*Geobacillus* virus E3]	7.0e-112<br><br>6.0e-20	-	-	COG5529; COG5529; Pyocin large subunit [Secondary metabolites biosynthesis, transport and catabolism].(98.21/1.70e-05)	replication initiator

								PF06970.12; RepA_N ; Replication initiator protein A (RepA) N-terminus(98.01/2.60e-05)<br><br>PF04492.14; Phage_rep_O ; Bacteriophage replication protein O (97.68/6.60e-04)	
24	16498	16920	+	hp	hp	-	-	COG2909; MalT; ATP-, maltotriose- and DNA-dependent transcriptional regulator MalT [Transcription].(98.49/1.00e-06)<br><br>d1p4wa_; a.4.6.2 (A:) Transcriptional regulator RcsB {*Erwinia amylovora* [TaxId: 552]}(98.27/5.20e-05)<br><br>d1l3la1; a.4.6.2 (A:170-234) Quorum-sensing transcription factor TraR, C-terminal domain {*Agrobacterium tumefaciens* [TaxId: 358]}(98.16/6.00e-05)	HTH domain containing transcriptional regulator
25	16989	17336	+	hp	hp	-	-	('-', '-')	hp
26	17336	17647	+	hp	hp	-	-	('-', '-')	hp
27	17653	17955	+	putative DNA binding protein [*Bacillus* phage PBC2]<br><br>DNA-binding protein [*Bacillus* phage Izhevsk]<br><br>integration host factor subunit alpha [*Bacillus* phage vB_BcoS-136]	4.0e-63<br>4.0e-50<br>4.0e-40	Bac_DNA_binding (35-94)<br><br>PRK10664 (1-94)	7.50e-07<br><br>8.60e-06	d1mula_; a.55.1.1 (A:) HU protein {*Escherichia coli* [TaxId: 562]}(99.9/2.30e-22)<br><br>d1p71a_; a.55.1.1 (A:) HU protein {Anabaena sp. [TaxId: 1167]}(99.9/4.50e-22)<br><br>d4p3va_; a.55.1.1 (A:) HU protein {*Escherichia coli*, beta-isoform [TaxId: 562]}(99.9/8.90e-22)	DNA-binding protein HU-beta

28	17975	19192	+	putative PhoH-like protein [*Bacillus* phage PBC2]<br><br>phosphate starvation-inducible protein [*Bacillus* phage pW2]<br><br>PhoH2 superfamily RNA helicase-ribonuclease [*Bacillus* phage Izhevsk]	0.0e+00<br><br>0.0e+00<br><br>0.0e+00	YlaK (3-405)<br><br>PhoH (209-399)<br><br>PRK10536 (204-356)	3.68e-32<br><br>2.61e-19<br><br>3.12e-11	COG1875; YlaK; Predicted ribonuclease YlaK, contains NYN-type RNase and PhoH-family ATPase domains [General function prediction o(100.0/3.50e-38)<br><br>COG1702; PhoH; Phosphate starvation-inducible protein PhoH, predicted ATPase [Signal transduction mechanisms].(99.82/9.40e-19)<br><br>PF02562.17; PhoH ; PhoH-like protein(99.81/8.60e-18)	PhoH2 superfamily RNA helicase-ribonuclease
29	20425	20628	+	hp	hp	YonK(2-56)	9.18e-04	d2h4oa1; d.368.1.1 (A:2-63) Uncharacterized protein YonK {*Bacillus subtilis* [TaxId: 1423]}(99.95/3.50e-27)<br><br>PF09642.11; ; YonK ; YonK protein(99.94/4.90e-26)	YonK-like protein
30	20640	21884	+	putative metallophophatase [*Bacillus* phage PBC2]<br><br>metallophosphatase [*Bacillus* phage Izhevsk]<br><br>putative metallophophatase/DNA polymerase [*Bacillus* phage pW2]	0.0e+00<br><br>0.0e+00<br><br>0.0e+00	MPP_YfcE (313-377)	1.95e-04	d2a22a1; d.159.1.7 (A:4-196) Vacuolar protein sorting 29, VPS29 {*Cryptosporidium parvum* [TaxId: 5807]}(99.73/6.90e-16)<br><br>d3ck2a1; d.159.1.7 (A:1-173) Uncharacterized protein SP1879 {*Pneumococcus* (*Streptococcus pneumoniae*) [TaxId: 1313]}(99.7/1.60e-15)<br>d1su1a_; d.159.1.7 (A:) Phosphodiesterase yfcE {*Escherichia coli* [TaxId: 562]}(99.62/4.80e-15)	metallophosphatase

31	22053	22952	+	putative small terminase [*Bacillus* phage Izhevsk]<br><br>YonG-like protein [*Brevibacillus* phage Sundance]	0.0e+00<br><br>2.0e-58	-	-	PF12898.8; Stc1; Stc1 domain (71.14/2.50e+00)	putative terminase, small subunit
32	22933	24621	+	putative terminase ATPase subunit [*Bacillus* phage PBC2]<br><br>putative terminase ATPase subunit [*Bacillus* phage pW2]<br><br>protein YonF [*Lactococcus* phage AM1]	0.0e+00<br><br>0.0e+00<br><br>0.0e+00	-	-	PF03354.16; terminase_1 ; Phage Terminase (100.0/2.30e-33)<br><br>COG5323; COG5323; Large terminase phage packaging protein [Mobilome: prophages, transposons].(100.0/9.10e-32)<br><br>COG4373; COG4373; Mu-like prophage FluMu protein gp28 [Mobilome: prophages, transposons].(100.0/6.80e-31)	terminase, large subunit
33	24636	26162	+	putative structural protein [*Bacillus* phage PBC2]<br><br>putative structural protein [*Bacillus* phage phiKir1]<br><br>portal protein [*Bacillus* phage Izhevsk]	0.0e+00<br><br>0.0e+00<br><br>0.0e+00	-	-	COG5511; COG5511; Bacteriophage capsid protein [Mobilome: prophages, transposons].(99.89/5.10e-21)<br><br>COG4695; BeeE; Phage portal protein BeeE [Mobilome: prophages, transposons].(99.67/8.20e-15)<br><br>PF05136.14; ; Phage_portal_2 ; Phage portal protein, lambda family (99.61/1.20e-14)	portal protein
34	26180	27568	+	putative prohead protease [*Bacillus* phage Izhevsk]<br><br>structural protein [*Lactococcus* phage WRP3]	0.0e+00<br><br>3.0e-43	-	-	PF09979.10; ; DUF2213 ; Uncharacterized protein conserved in bacteria (DUF2213)(94.96/4.30e-01)	putative prohead protease

				structural protein [*Lactococcus* phage AM1]	2.0e-42			PF05929.12; Phage_GPO ; Phage capsid scaffolding protein (GPO) serine peptidase (94.31/1.5)<br><br>PF03420.14; Peptidase_S77 ; Prohead core protein serine protease (93.99/3.8)	
35	27585	28091	+	structural protein [*Lactococcus* phage WRP3]<br><br>putative structural protein [*Lactococcus* phage phiL47]<br><br>putative phage structural protein [*Lactococcus* phage 949]	5.0e-25<br><br>2.0e-24<br><br>2.0e-24	-	-	('-', '-')	hp
36	28122	29180	+	structural protein [*Lactococcus* phage AM4]<br><br>structural protein [*Lactococcus* phage WRP3]<br><br>structural protein [*Lactococcus* phage AM1]	3.0e-79<br><br>6.0e-79<br><br>1.0e-78	-	-	d2fsya1; d.183.1.1 (A:104-383) Major capsid protein gp5 {Bacteriophage HK97 [TaxId: 37554]}(98.0/1.50e-04)<br><br>COG4653 COG4653; Predicted phage phi-C31 gp36 major capsid-like protein [Mobilome: prophages, transposons]. (96.75/0.22)<br><br>PF05065.14; Phage_capsid ; Phage capsid family (94.62/1.8)	major capsid protein
37	29279	29983	+	putative AcrB/AcrD/AcrF family protein [*Brevibacillus* phage Sundance]	2.0e-17	-	-	('-', '-')	hp
38	29996	30457	+	putative structural protein [*Bacillus* phage PBC2]	8.0e-93<br>1.0e-55<br>4.0e-22	-	-	PF11650.9; P22_Tail-4 ; P22 tail accessory factor (27.62/330)	hp

				putative structural protein [*Bacillus* phage pW2]<br><br>putative structural protein [*Lactococcus* phage phiL47]					
39	30454	30771	+	hp	hp	-	-	('-', '-')	hp
40	30774	31529	+	putative structural protein [*Bacillus* phage PBC2]<br><br>head completion protein [*Bacillus* phage Izhevsk]<br><br>putative structural protein [*Bacillus* phage pW2]	2.0e-159<br><br>1.0e-130<br><br>3.0e-125	-	-	COG5614; COG5614; Bacteriophage head-tail adaptor [Mobilome: prophages, transposons].(94.1/7.70e-01)<br><br>PF05354.12; Phage_attach ; Phage Head-Tail Attachment (93.82/1.3)<br><br>d2kx4a_ b.106.1.2 (A:) Tail attachment protein gpF3 {Bacteriophage lambda [TaxId: 10710]} (91.86/2.4)	head completion protein
41	31529	32026	+	putative structural protein [*Bacillus* phage pW2]<br><br>structural protein [*Lactococcus* phage WRP3]<br><br>structural protein [*Lactococcus* phage AM4]	4.0e-62<br><br>2.0e-24<br><br>9.0e-24	-	-	PF06526.13; ; DUF1107 ; Protein of unknown function (DUF1107)(73.13/4.30e+00)<br><br>PF09646.11; Gp37 ; Gp37 protein (50.27/120)<br><br>PF06141.12; Phage_tail_U ; Phage minor tail protein U (34.98/210)	putative tail terminator protein
42	32033	32893	+	putative tail assembly chaperone [*Bacillus* phage Izhevsk]	3.0e-89	-	-	PF10109.10; Phage_TAC_7 ; Phage tail assembly chaperone proteins, E, or 41 or 14 (14.96/310)	putative tail assembly chaperone
43	32940	33695	+	putative structural protein [*Bacillus* phage PBC2]<br><br>tail tube protein [*Bacillus* phage Izhevsk]	2.0e-127<br><br>5.0e-120	-	-	PF09343.11; ; DUF2460 ; Conserved hypothetical protein 2217 (DUF2460)(85.26/4.10e+00)	tail tube protein

				putative structural protein [*Bacillus* phage pW2]	1.0e-114			PF04630.13; Phage_TTP_1 ; Phage tail tube protein (79.15/33)<br><br>PF04630.13; Phage_TTP_1 ; Phage tail tube protein (74.25/18)<br><br>PF06488.12; L_lac_phage_MSP ; Phage tail tube protein (43.55/330)	
44	33871	34392	+	hp	hp	-	-	('-', '-')	hp
45	34361	34843	+	putative structural protein [*Bacillus* phage PBC2]<br><br>tail completion protein [*Bacillus* phage Izhevsk]<br><br>structural protein [*Lactococcus* phage AM4]	2.0e-98<br><br>4.0e-39<br><br>4.0e-12	-	-	COG5005; COG5005; Mu-like prophage protein gpG [Mobilome: prophages, transposons]. (96.57/4.40e-02)	tail completion protein
46	34859	35836	+	putative site specific recombinase [*Bacillus* phage phiKir1]<br><br>putative site specific recombinase [*Bacillus* phage PBC2]<br><br>putative site specific recombinase [*Bacillus* phage pW2]	0.0e+00<br><br>0.0e+00<br><br>1.0e-155	recomb_XerC (27-312)<br><br>DNA_BRE_C (140-313)	1.72e-28<br><br>7.61e-24	COG4973; XerC; Site-specific recombinase XerC [Replication, recombination and repair]. (100.0/2.30e-33)<br><br>COG4974; XerD; Site-specific recombinase XerD [Replication, recombination and repair]. (100.0/9.50e-32)<br><br>COG4342; COG4342; Intergrase/Recombinase [Mobilome: prophages, transposons].(99.93/1.30e-24)	Xer family site-specific tyrosine recombinase
47	35913	45080	+	tape measure protein [*Bacillus* phage Izhevsk]	0.0e+00	tape_meas_TP901 (636-981)	4.87e-41	COG5280; YqbO; Phage-related minor tail protein [Mobilome: prophages, transposons].(99.43/2.50e-08)	tape measure protein

				putative tail tape measure protein [*Bacillus* phage pW2]<br><br>putative tail tape measure protein [*Bacillus* phage PBC2]	0.0e+00<br><br>0.0e+00	PhageMin_Tail (676-877)<br><br>SH3_3 (2896-2951)	2.50e-18<br><br>3.80e-10	COG5283; COG5283; Phage-related tail protein [Mobilome: prophages, transposons].(99.43/3.20e-07)<br><br>PF10145.10; PhageMin_Tail ; Phage-related minor tail protein(98.73/1.60e-06)	
48	45124	45894	+	tail protein [*Bacillus* phage Izhevsk]<br><br>tail protein [*Bacillus* phage pW2]<br><br>YomH-like protein [*Brevibacillus* phage Sundance]	6.0e-138<br><br>1.0e-129<br><br>4.0e-35	YomH (9-255)<br><br>Sipho_tail (40-255)	2.98e-17<br><br>2.61e-07	COG4722; YomH; Phage-related protein [Mobilome: prophages, transposons]. (99.97/6.30e-29)<br><br>PF05709.12; Sipho_tail; Phage tail protein (99.83/1.40e-17)<br><br>PF16774.6; Baseplate; Baseplate protein (95.57/0.91)	distal tail protein
49	45957	46442	+	putative cell wall-associated hydrolase [*Bacillus* phage pW2]<br><br>putative cell wall-associated hydrolase [*Bacillus* phage vB_BcoS-136]<br><br>putative permuted papain-like amidase enzyme [*Bacillus* phage vB_BsuM-Goe3]	4.0e-74<br><br>5.0e-62<br><br>2.0e-33	Peptidase_C92 (3-161)<br><br>PRK10030 (3-134)	1.30e-07<br><br>5.75e-06	d2if6a1; d.3.1.21 (A:19-200) Hypothetical protein YiiX {*Escherichia coli* [TaxId: 562]} (99.93/3.50e-24)<br><br>cd13955; DUF830; Orthopoxvirus protein of unknown function (DUF830). (99.92/3.10e-23)<br><br>PF05708.13; Peptidase_C92; Permuted papain-like amidase enzyme, YaeF/YiiX, C92 family (99.91/8.00e-23)	permuted papain-like amidase
50	46459	49119	+	putative tail endopeptidase [*Bacillus* phage Izhevsk]<br><br>minor structural protein [*Bacillus* phage pW2]	0.0e+00<br><br>0.0e+00	put_anti_recept (43-254)<br><br>Prophage_tail (387-500)	5.64e-09<br>1.75e-08<br>1.01e-05	COG4926; PblB; Phage-related protein [Mobilome: prophages, transposons]. (99.97/9.50e-29)<br>PF06605.12; ; Prophage_tail ; Prophage endopeptidase tail(99.56/1.50e-13)<br>PF14594.7; ; Sipho_Gp37 ; Siphovirus	tail endopeptidase

				putative tail protein [*Lactococcus* phage phiL47]	1.0e-128	Prophage_tail (44-253)		ReqiPepy6 Gp37-like protein(99.5/8.60e-12)	
51	49151	50890	+	putative tail protein [*Bacillus* phage pW4]<br><br>putative tail protein [*Bacillus* phage PBC4]<br><br>minor structural protein [*Bacillus* phage pW2]	1.0e-146<br><br>3.0e-144<br><br>2.0e-106	-	-	KOG4194 Membrane glycoprotein LIG-1 [Signal transduction mechanisms] (70.75/92)	hp
52	51025	55065	+	putative receptor-binding protein [*Bacillus* phage Izhevsk]<br><br>tail fiber protein [*Bacillus* phage pW2]<br><br>putative tail fiber [*Bacillus* phage phiNIT1]	0.0e+00<br><br>0.0e+00<br><br>1.0e-95	-	-	d1h6ya_; b.18.1.7 (A:) Xylan-binding domain {*Clostridium thermocellum* [TaxId: 1515]} (97.32/1.40e-02)<br><br>d1cx1a1; b.18.1.14 (A:3-153) Cellulose-binding domain of cellulase C {*Cellulomonas fimi* [TaxId: 1708]} (96.91/2.80e-02)<br><br>d1guia_; b.18.1.14 (A:) Carbohydrate binding module from laminarinase 16A {*Thermotoga maritima* [TaxId: 2336]} (96.59/9.70e-02)	receptor-binding protein
53	55089	57830	+	endosialidase [*Bacillus* phage Izhevsk]<br><br>minor tail protein [*Bacillus* phage pW2]<br><br>chaperone domain of endosialidase [*Bacillus* phage Kioshi]	0.0e+00<br><br>0.0e+00<br><br>0.0e+00	DUF859 (7-600)<br><br>Peptidase_S74 (811-861)	1.79e-22<br><br>1.68e-04	PF05895.13; DUF859; Siphovirus protein of unknown function (DUF859) (100.0/1.50e-67)<br><br>KOG3661; Uncharacterized conserved protein [Function unknown] (99.38/1.60e-12)<br><br>PF17253.3; DUF5320; Family of unknown function (DUF5320)(94.43/7.30e-02)	endosialidase

54	57833	58330	+	hp	hp	-	-	PF14301.7; DUF4376; Domain of unknown function (DUF4376) (99.38/4.70e-11)<br><br>PF10653.10; Phage-A118_gp45; Protein gp45 of Bacteriophage A118 (25.33/69)	hp
55	58358	58519	+	hp	hp	-	-	COG2960; YqiC; Uncharacterized conserved protein YqiC, BMFP domain [Function unknown]. (86.94/6.20e+00)<br>PF10224.10; DUF2205; Short coiled-coil protein (76.33/12)<br>KOG3650 Predicted coiled-coil protein [General function prediction only] (69.77/17)<br>PF17594.3; GP57; Phage Tail fiber assembly helper gene product 57 (52.15/76)	putative tail fiber chaperone
56	58597	59532	+	N-acetylmuramoyl-L-alanine amidase [*Bacillus* phage Izhevsk]<br><br>putative alanine amidase/endolysin [*Bacillus* phage pW2]<br><br>putative alanine amidase [*Bacillus* phage PBC2]	0.0e+00<br><br>0.0e+00<br><br>0.0e+00	CwlA (3-161)<br><br>SH3b (252-304)	1.35e-62<br><br>2.40e-04	d1yb0a1; d.118.1.1 (A:1-157) N-acetylmuramoyl-L-alanine amidase PlyG {Anthrax *Bacillus* (*Bacillus* anthracis) [TaxId: 1392]} (99.83/2.80e-18)<br><br>COG5632; CwlA; N-acetylmuramoyl-L-alanine amidase CwlA [Cell wall/membrane/envelope biogenesis]. (99.71/2.20e-15)<br><br>d2cb3a1; d.118.1.1 (A:174-344) Peptidoglycan-recognition protein-LE {Fruit fly (Drosophila melanogaster) [TaxId: 7227]} (99.58/2.90e-14)	N-acetylmuramoyl-L-alanine amidase
57	59603	59929	+	hp	hp	-	-	COG3105; YhcB; Uncharacterized membrane-anchored protein YhcB,	putative holin

								DUF1043 family [Function unknown]. (98.27/1.90e-04)<br><br>COG4942; EnvC; Septal ring factor EnvC, activator of murein hydrolases AmiA and AmiB [Cell cycle control, cell division, chromoso(98.16/4.10e-04)<br><br>COG3883 CwlO1; Uncharacterized N-terminal domain of peptidoglycan hydrolase CwlO [Function unknown]. (97.6/0.014)<br><br>PF16082.6; Phage_holin_2_4; Bacteriophage holin family, superfamily II-like (95.3/0.077)	
58	59988	60326	+	holin [*Bacillus* phage Izhevsk]<br><br>putative holin [*Bacillus* phage PBC2]<br><br>holin [*Bacillus* phage vB_BcoS-136]	4.0e-52<br><br>6.0e-51<br><br>4.0e-33	Phage_holin_6_1 (8-103)	5.00e-11	PF09682.11; hage_holin_6_1; Bacteriophage holin of superfamily 6 (Holin_LLH)(99.89/6.00e-22)	holin
59	60346	60525	+	hp	hp	-	-	COG4873; YkvS; Uncharacterized protein YkvS, DUF2187 family [Function unknown]. (96.76/7.70e-03)<br><br>PF09953.10; DUF2187; Uncharacterized protein conserved in bacteria (DUF2187) (96.78/1.10e-02)<br><br>cd06085; KOW_Spt5_5; KOW domain of Spt5, repeat 5. Spt5, an eukaryotic	hp

								ortholog of NusG, contains multiple KOW motifs at its C-te(96.31/3.10e-02)	
60	60539	61213	+	putative Ig-like domain-containing protein [*Bacillus* phage Izhevsk]	2.0e-133	-	-	PF13750.7; Big_3_3; Bacterial Ig-like domain (group 3) (95.32/8.20e-01)	putative Ig-like domain-containing protein
61	61265	61720	+	putative Ig-like domain-containing protein [*Bacillus* phage Izhevsk]	9.0e-82	-	-	PF15418.7; DUF4625; Domain of unknown function (DUF4625) (96.38/1.80e-01)<br><br>PF13750.7; Big_3_3; Bacterial Ig-like domain (group 3) (76.35/25)	putative Ig-like domain-containing protein
62	61726	62010	+	hp	hp	-	-	COG2373; YfaS; Uncharacterized conserved protein YfaS, alpha-2-macroglobulin family [General function prediction only]. (98.91/7.00e-08)<br><br>PF01835.20; MG2; MG2 domain(98.87/1.80e-07)<br><br>KOG1366; Alpha-macroglobulin [Posttranslational modification, protein turnover, chaperones] (98.77/2.80e-07)	hp
63	62023	62985	+	concanavalin A-like lectin/glucanase superfamily protein [*Bacillus* phage Izhevsk]<br><br>putative concanavalin A-like lectin/glucanases superfamily protein [Prokaryotic dsDNA virus sp.]	0.0e+00<br><br>1.0e-05	Laminin_G_3 (141-210)	1.72e-10	d1ms9a1; b.29.1.15 (A:409-632) Trypanosoma sialidase, C-terminal domain {*Trypanosoma cruzi* [TaxId: 5693]} (99.1/3.10e-07)<br><br>d1n1ta1; b.29.1.15 (A:413-634) *Trypanosoma sialidase*, C-terminal domain {*Trypanosoma rangeli* [TaxId: 5698]} (99.05/8.90e-07)	Concanavalin A-like lectin/glucanases superfamily protein

				Concanavalin A-like lectin/glucanases superfamily [uncultured *Caudovirales* phage]	1.0e-03			d1a8da1; b.29.1.6 (A:1-247) *Tetanus neurotoxin* {*Clostridium tetani* [TaxId: 1513]} (98.9/4.70e-06)	
64	63065	63592	+	hp	hp	-	-	PF17726.2; DpnI_C; Dam-replacing HTH domain (96.18/4.10e-02)	HTH domain containing protein
65	64127	63648	-	-	-	-	-	('-', '-')	hp
66	64785	64147	-	-	-	-	-	('-', '-')	hp
67	65367	64789	-	holliday junction resolvase [*Bacillus* phage pW2]<br><br>holliday-junction resolvase [*Bacillus* phage Izhevsk]<br><br>holliday junction resolvase [*Bacillus* phage vB_BcoS-136]	4.0e-66<br><br>2.0e-64<br><br>1.0e-48	RuvC (9-169)<br><br>ruvC (55-168)	8.32e-22<br><br>4.95e-04	d4ep4a_; c.55.3.6 (A:) RuvC resolvase {Thermus thermophilus [TaxId: 300852]} (99.93/3.10e-23)<br><br>d1hjra_; c.55.3.6 (A:) RuvC resolvase {*Escherichia coli* [TaxId: 562]} (99.92/1.40e-22)<br><br>COG0817; RuvC; Holliday junction resolvasome RuvABC endonuclease subunit [Replication, recombination and repair]. (99.91/1.60e-22)	Holliday junction resolvase RuvC
68	65606	65442	-	hp	hp	-	-	d1imla1; g.39.1.3 (A:1-28) Cysteine-rich (intestinal) protein, CRP, CRIP {Rat (Rattus rattus) [TaxId: 10117]} (96.89/6.40e-04)<br><br>d1b8ta1; g.39.1.3 (A:1-35) Cysteine-rich (intestinal) protein, CRP, CRIP {Chicken (Gallus gallus) [TaxId: 9031]} (96.55/1.90e-03)<br><br>d1zfoa_; g.39.1.4 (A:) LASP-1 {Pig (Sus scrofa) [TaxId: 9823]} (96.49/2.40e-03)	hp

69	69064	65690	-	DNA polymerase III alpha subunit [*Bacillus* phage PBC2]<br><br>DNA polymerase III subunit alpha [*Bacillus* phage vB_BcoS-136]<br><br>DNA polymerase III [*Clostridioides* phage LIBA6276]	0.0e+00<br><br>0.0e+00<br><br>0.0e+00	DnaE (4-958)<br><br>dnaE (1-877)<br><br>DNA_pol3_alpha (330-581)	2.21e-137<br><br>4.82e-118<br><br>4.38e-61	COG0587; DnaE; DNA polymerase III, alpha subunit [Replication, recombination and repair]. (100.0/5.70e-158)<br><br>COG2176; PolC; DNA polymerase III, alpha subunit (gram-positive type) [Replication, recombination and repair]. (100.0/7.20e-125)<br><br>PF07733.13; DNA_pol3_alpha; Bacterial DNA polymerase III alpha NTPase domain (100.0/1.50e-45)	DNA polymerase III alpha subunit
70	69575	69078	-	hp	hp	-	-	COG5475; YodC; Uncharacterized conserved protein YodC, DUF2158 family [Function unknown]. (84.07/1.20e+00)	hp
71	70278	69658	-	putative tymidine kinase [*Bacillus* phage PBC2]<br><br>thymidine kinase [*Bacillus* phage Izhevsk]<br><br>putative thymidine kinase [*Bacillus* phage pW2]	8.0e-136<br><br>6.0e-109<br><br>8.0e-100	PRK04296 (1-191)	1.49e-29	COG1435; Tdk; Thymidine kinase [Nucleotide transport and metabolism]. (99.95/4.80e-26)<br><br>KOG3125; Thymidine kinase [Nucleotide transport and metabolism] (99.95/2.30e-25)<br><br>PF00265.19; TK; Thymidine kinase (99.9/1.30e-21)	thymidine kinase
72	70587	70288	-	hp	hp	-	-	PF09435.11; DUF2015; Fungal protein of unknown function (DUF2015) (76.6/5.70e+00)	hp
73	71152	70574	-	putative 5'-nucleotidase [*Bacillus* phage Izhevsk]<br><br>putative phosphohydrolase [*Bacillus* phage pW2]	1.0e-89<br><br>4.0e-83	YfbR (41-115)<br><br>HD_2 (38-125)	5.84e-09<br><br>(7.01e-07	d2gz4a1; a.211.1.1 (A:6-205) Hypothetical protein Atu1052 {*Agrobacterium tumefaciens* [TaxId: 358]} (100.0/1.00e-37)	5'-nucleotidase

				putative phosphohydrolase [*Brevibacillus* phage Sundance]	2.0e-30			KOG3197; Predicted hydrolases of HD superfamily [General function prediction only] (99.61/1.90e-14)<br><br>d1xx7a1; a.211.1.1 (A:2-172) Oxetanocin-like protein PF0395 {*Pyrococcus furiosus* [TaxId: 2261]} (99.44/3.50e-12)<br><br>d2para_ a.211.1.1 (A:) 5'-nucleotidase YfbR {*Escherichia coli* [TaxId: 562]} (99.31/1e-10)	
74	71373	71152	-	hp	hp	-	-	PF13113.7; DUF3970; Protein of unknown function (DUF3970)(86.08/3.00e+00)	hp
75	72120	71452	-	ATP-dependent Clp protease proteolytic subunit 2 [*Bacillus* phage pW2]<br><br>ATP-dependent Clp protease proteolytic subunit 2 [*Bacillus* phage vB_BcoS-136]<br><br>ATP-dependent Clp protease proteolytic subunit 1 [*Bacillus* phage pW2]	1.0e-83<br><br>2.0e-83<br><br>1.0e-27	S14_ClpP_2 (7-178)<br><br>CLP_protease (7-174)	5.48e-30<br><br>4.47e-29	KOG0840; ATP-dependent Clp protease, proteolytic subunit [Posttranslational modification, protein turnover, chaperones] (99.92/6.60e-23)<br><br>d2zl4a_; c.14.1.1 (A:) Clp protease, ClpP subunit {*Helicobacter pylori* [TaxId: 210]} (99.91/1.10e-21)<br><br>d2f6ia1; c.14.1.1 (A:179-366) Clp protease, ClpP subunit {Malaria parasite (*Plasmodium falciparum*) [TaxId: 5833]} (99.9/3.30e-21)	ATP-dependent Clp protease proteolytic subunit
76	72696	72133	-	putative dihydrofolate reductase [*Bacillus* phage PBC2]	2.0e-98<br><br>9.0e-77	DHFR_1 (3-164)<br><br>FolA (3-165)	3.53e-65<br><br>3.13e-46	d4psya_; c.71.1.1 (A:) Dihydrofolate reductase, prokaryotic type {*Escherichia coli* [TaxId: 562]} (99.94/7.20e-25)	dihydrofolate reductase

				dihydrofolate reductase [*Bacillus* phage vB_BcoS-136]<br><br>putative dihydrofolate reductase [*Bacillus* phage pW2]	1.0e-64			d3dfra_; c.71.1.1 (A:) Dihydrofolate reductase, prokaryotic type {*Lactobacillus casei* [TaxId: 1582]} (99.94/6.20e-24)<br><br>d1j3ka_; c.71.1.1 (A:) Bifunctional enzyme dihydrofolate reductase-thymidylate synthase, DFR domain (99.92/5.40e-23)	
77	72937	72698	-	hp	hp	-	-	('-', '-')	hp
78	73782	72949	-	thymidylate synthase [*Bacillus* phage Izhevsk]<br><br>thymidylate synthase [*Bacillus* phage pW2]<br><br>thymidylate synthase [*Psychrobacillus* phage Perkons]	0.0e+00<br><br>5.0e-154<br><br>7.0e-114	Thymidylat_synt (8-275)<br><br>TS_Pyrimidine_HMase (7-227)<br><br>thym_sym (8-266)	1.11e-75<br><br>1.06e-45<br><br>4.32e-33	d4iw5a_; d.117.1.1 (A:) Thymidylate synthase {*Escherichia coli* [TaxId: 562]} (100/6.00e-56)<br><br>d1bkpa_; d.117.1.1 (A:) Thymidylate synthase {*Bacillus subtilis* [TaxId: 1423]} (100/6.90e-56)<br><br>d1j3kc_; d.117.1.1 (C:) Bifunctional enzyme dihydrofolate reductase-thymidylate synthase, TS domain {Malaria parasite (*Plasmodium*(100/9.80e-56)	thymidylate synthase
79	74185	73811	-	hp	hp	-	-	d1z05a1; a.4.5.63 (A:10-80) Transcriptional regulator VC2007 N-terminal domain {*Vibrio cholerae* [TaxId: 666]} (98.5/4.10e-07)<br>d3bp8a1; a.4.5.63 (A:11-81) Mlc protein N-terminal domain {*Escherichia coli* [TaxId: 562]} (98.48/6.10e-07)<br>COG2345; COG2345; Predicted transcriptional regulator, ArsR family [Transcription]. (98.49/6.30e-07)	putative transcriptional regulator
80	74676	74197	-	transposase [*Bacillus* phage Izhevsk]	1.0e-50	-	-	PF01710.17; HTH_Tnp_IS630; Transposase (98.76/7.20e-07)	HTH domain containing protein

								COG2826; Tra8; Transposase and inactivated derivatives, IS30 family [Mobilome: prophages, transposons]. (98.55/2.90e-06)<br><br>COG1675; TFA1; Transcription initiation factor IIE, alpha subunit [Transcription]. (98.03/3.60e-06)	
81	75059	74691	-	putative 5'-nucleotidase [*Bacillus* phage Izhevsk]<br><br>HAD-like domain containing protein [*Enterococcus* phage vB_EfaM_A2]<br><br>HAD-like family phosphatase [*Enterococcus* phage iF6]	8.0e-58<br><br>2.0e-17<br><br>4.0e-17	-	-	d2obba1; c.108.1.25 (A:1-122) Hypothetical protein BT0820 {*Bacteroides thetaiotaomicron* [TaxId: 818]} (99.71/4.70e-16)<br><br>PF06189.13; 5-nucleotidase; 5'-nucleotidase (99.1/7.70e-10)<br><br>cd07499; HAD_CBAP; molecular class B acid phosphatases, similar to *Escherichia coli* AphA. class B acid phosphatases (CBAPs) have (99.05/4.20e-09)	5'-nucleotidase
82	75521	75072	-	putative signal peptide-containing lipoprotein [*Bacillus* phage Izhevsk]	6.0e-12	-	-	PF16339.6; DUF4969; Domain of unknown function (DUF4969) (95.11/3.70e-02)<br>COG5567 YifL; Predicted small periplasmic lipoprotein YifL (function unknown0 [Function unknown]. (94.01/0.11)	signal peptide-containing lipoprotein
83	75706	75518	-	gp186 [*Bacillus* phage W.Ph.]<br><br>gp33 [*Bacillus* phage TP21-L]	4.0e-06<br><br>2.0e-04	-	-	('-', '-')	hp

84	76253	75810	-	GatB/YqeY domain-containing protein [*Bacillus* phage Izhevsk]<br><br>glutamyl-tRNA amidotransferase [*Xanthomonas* phage XacN1]<br><br>gp509 [*Bacillus* virus G]	5.0e-34<br><br>9.0e-12<br><br>4.0e-11	YqeY (5-141)	1.69e-19	COG1610; YqeY; Uncharacterized conserved protein YqeY [Function unknown] (99.96/2.80e-27)<br><br>d1ng6a_; a.182.1.1 (A:) Hypothetical protein YqeY {*Bacillus subtilis* [TaxId: 1423]} (99.96/4.10e-27)<br><br>PF09424.11; YqeY; Yqey-like protein (99.95/7.70e-26)	GatB/YqeY domain containing protein
85	76519	76322	-	hp	hp	-	-	PF10957.9; Spore_Cse60; Sporulation protein Cse60(99.75/2.00e-17)<br><br>PF10750.10; DUF2536; Protein of unknown function (DUF2536) (98.03/1.10e-04)	sporulation protein Cse60
86	76706	76512	-	hp	hp	-	-	COG2919; FtsB; Cell division protein FtsB [Cell cycle control, cell division, chromosome partitioning] (92.28/5.00e-01)	putative cell division protein FtsB
87	78552	76816	-	nicotinate phosphoribosyltransferase [*Bacillus* phage Izhevsk]<br><br>nicotinamide phosphoribosyl transferase [*Bacillus* phage vB_BcoS-136]<br><br>nicotinamide phosphoribosyl transferase [*Bacillus* phage PBC2]	0.0e+00<br><br>0.0e+00<br><br>0.0e+00	nadV (1-578)<br><br>PBEF_like (12-522)<br><br>NAPRTase (187-560)	0e+00<br><br>0e+00<br><br>1.44e-33	cd01569; PBEF_like; pre-B-cell colony-enhancing factor (PBEF)-like. The mammalian members of this group of nicotinate phosphoribo (100.0/6.00e-68)<br><br>cd01401; PncB_like; Nicotinate phosphoribosyltransferase (NAPRTase), related to PncB. Nicotinate phosphoribosyltransferase cataly (100.0/2.40e-51)<br><br>KOG2511; Nicotinic acid phosphoribosyltransferase [Coenzyme	nicotinate phosphoribosyl transferase

								transport and metabolism] (100.0/9.70e-48)	
88	78766	78572	-	hp	hp	-	-	PF09629.11; YorP; YorP protein (98.69/3.90e-07)<br>COG5475; YodC; Uncharacterized conserved protein YodC, DUF2158 family [Function unknown]. (97.61/2.30e-03)<br>PF18354.2; SH3_18; CarS bacterial SH3 domain (97.15/4.00e-03)	YorP-like protein
89	79025	78768	-	hp	hp	-	-	PF09629.11; YorP; YorP protein (98.07/3.00e-05)	YorP-like protein
90	79896	79012	-	phosphoribosylpyrophosphate synthetase [*Bacillus* phage Izhevsk]<br><br>ribose-phosphate pyrophosphokinase [*Bacillus* phage vB_BcoS-136]<br><br>putative ribose-phosphate pyrophosphokinase [*Bacillus* phage BPS13]	2.0e-93<br><br>6.0e-91<br><br>3.0e-71	ribP_PPkin(8-276)<br><br>PRTases_typeI(133-264)<br><br>PrsA(9-286)	5.15e-22<br><br>3.66e-07<br><br>1.57e-21	KOG1448; Ribose-phosphate pyrophosphokinase [Nucleotide transport and metabolism, Amino acid transport and metabolism] (100.0/1.50e-42)<br><br>KOG1503; Phosphoribosylpyrophosphate synthetase-associated protein [Amino acid transport and metabolism, Nucleotide transport and (100.0/2.30e-42)<br><br>COG0462; PrsA; Phosphoribosylpyrophosphate synthetase [Nucleotide transport and metabolism, Amino acid transport and metabolism]. (100.0/3.90e-40)	phosphoribosylpyrophosphate synthetase
91	80137	79898	-	hp	hp	-	-	('-', '-')	hp
92	80592	80179	-	hp	hp	-	-	d1kfia4; d.129.2.1 (A:444-572) Exocytosis-sensitive phosphoprotein, pp63/parafusin {*Ciliate (Paramecium tetraurelia*) [TaxId: 5888(86.89/4.20e+00)	hp
93	80887	80594	-	hp	hp	-	-	('-', '-')	hp
94	81153	80887	-	hp	hp	-	-	('-', '-')	hp

95	82465	81158	-	DNA ligase [*Bacillus* phage PBC2]<br><br>DNA ligase, ATP-dependent [*Bacillus* phage vB_BanS-Tsamsa]<br><br>DNA ligase [*Bacillus* phage Izhevsk]	0.0e+00<br><br>0.0e+00<br><br>0.0e+00	30<br>(5-426)<br><br>CDC9<br>(106-430)<br><br>Adenylation_kDNA_ligase_like<br>(139-335)	1.93e-41<br><br>4.22e-32<br><br>6.69e-32	KOG0967; ATP-dependent DNA ligase I [Replication, recombination and repair] (100.0/1.70e-57)<br><br>KOG0966; ATP-dependent DNA ligase IV [Replication, recombination and repair] (100.0/2.30e-56)<br><br>COG1793; CDC9; ATP-dependent DNA ligase [Replication, recombination and repair]. (100.0/1.90e-45)	ATP-dependent DNA ligase
96	83086	82484	-	ATP-dependent Clp protease proteolytic subunit 1 [*Bacillus* phage pW2]<br><br>putative Clp endopeptidase [*Bacillus* phage PBC2]<br><br>ATP-dependent Clp protease proteolytic subunit 1 [*Bacillus* phage vB_BcoS-136]	4.0e-87<br><br>6.0e-79<br><br>2.0e-64	S14_ClpP_2<br>(26-192)<br><br>ClpP<br>(55-200)<br><br>CLP_protease<br>(22-196)	6.42e-20<br><br>1.06e-16<br><br>1.78e-15	KOG0840; ATP-dependent Clp protease, proteolytic subunit [Posttranslational modification, protein turnover, chaperones] (99.93/1.80e-23)<br><br>d2zl4a_; c.14.1.1 (A:) Clp protease, ClpP subunit {*Helicobacter pylori* [TaxId: 210]} (99.92/8.90e-23)<br><br>d2f6ia1; c.14.1.1 (A:179-366) Clp protease, ClpP subunit {Malaria parasite (*Plasmodium falciparum*) [TaxId: 5833]} (99.92/2.10e-22)	ATP-dependent Clp protease proteolytic subunit
97	83419	83123	-	hp	hp	-	-	PF11305.9; DUF3107; Protein of unknown function (DUF3107) (94.92/2.20e-01)	hp
98	84424	83432	-	AAA family ATPase domain-containing protein [*Bacillus* phage Izhevsk]	9.0e-134<br><br>1.0e-125	COG4639<br>(7-141)<br><br>AAA_33<br>(9-136)	4.51e-20<br><br>5.89e-17<br><br>1.15e-09	COG4639; COG4639; Predicted kinase [General function prediction only]. (99.53/1.50e-12)	polynucleotide kinase

				polynucleotide kinase [*Bacillus* phage vB_BcoS-136]<br><br>polynucleotide kinase [*Bacillus* phage pW2]	6.0e-115	tRNA_CCA_actino (183-295)<br><br>selen_PSTK (10-144)	6.07e-09	d1knqa_; c.37.1.17 (A:) Gluconate kinase {*Escherichia coli* [TaxId: 562]} (99.54/1.90e-12)<br><br>KOG3062; RNA polymerase II elongator associated protein [General function prediction only] (99.44/3.00e-12)	
99	85755	84475	-	2'-5' RNA ligase [*Bacillus* phage PBC2]<br><br>RNA ligase [*Bacillus* phage vB_BcoS-136]<br><br>RNA ligase domain protein [*Vibrio* phage 2.275.O._10N.286.54.E11]	0.0e+00<br><br>0.0e+00<br><br>7.0e-78	RNA_lig_DRB0094 (5-186)<br><br>PHA02142 (5-114)	2.32e-13<br><br>9.70e-04	d1xdna_; d.142.2.4 (A:) RNA editing ligase MP52 {*Trypanosome* (*Trypanosoma brucei*) [TaxId: 5691]} (99.9/6.00e-23)<br><br>d1s68a_; d.142.2.4 (A:) RNA ligase 2, N-terminal domain {Bacteriophage T4 [TaxId: 10665]} (99.81/4.10e-19)<br><br>PF09511.11; RNA_lig_T4_1; RNA ligase (99.52/2.50e-13)	RNA ligase
100	86915	86055	-	putative subtilisin-like protein [*Bacillus* phage PBC2]<br><br>serine peptidase S8 [*Bacillus* phage Izhevsk]<br><br>putative subtilisin-like protein [*Bacillus* phage pW2]	1.0e-178<br><br>1.0e-170<br><br>5.0e-149	Peptidases_S8_Subtilisin_subset (40-264)<br><br>AprE (10-268)<br><br>PTZ00262 (41-286)	5.12e-83<br><br>1.87e-38<br><br>7.75e-21	d1r6va_; c.41.1.1 (A:) Fervidolysin {*Fervidobacterium pennivorans* [TaxId: 93466]} (100.0/9.90e-33)<br>d1supa_; c.41.1.1 (A:) Subtilisin {*Bacillus amyloliquefaciens*, Novo/BPN' [TaxId: 1390]} (100.0/4.00e-32)<br><br>d1gcia_; c.41.1.1 (A:) *Subtilisin* {*Bacillus lentus* [TaxId: 1467]} (100.0/5.40e-32)	subtilisin-like serine protease
101	87512	86922	-	putative nucleotidyltransferase [*Bacillus* phage Izhevsk]<br><br>gp109 [*Bacillus* virus G]<br><br>Polymerase, nucleotidyl transferase domain containing	1.0e-92<br>3.0e-61<br>5.0e-37	NT_KNTase_like (6-56)<br><br>YcgL (12-150)	1.23e-04<br><br>9.81e-04	PF10127.10; RlaP; RNA repair pathway DNA polymerase beta family(99.81/6.10e-19)<br><br>COG3541; YcgL; Predicted nucleotidyltransferase [General function prediction only].(99.67/2.00e-15)	nucleotidyltransferase

				protein [uncultured *Caudovirales* phage]				PF14540.7; NTF-like; Nucleotidyltransferase-like(98.82/1.50e-08)	
102	88167	87523	-	chaperone protein [*Bacillus* phage vB_BcoS-136]<br><br>chaperone protein DnaJ [uncultured *Caudovirales* phage]<br><br>DnaJ DnaJ-class molecular chaperone with C-terminal Zn finger domain [uncultured *Caudovirales* phage]	7.0e-24<br>1.0e-09<br>2.0e-07	DnaJ (167-208)<br><br>djlA (133-192)	2.52e-12<br><br>3.28e-10	KOG0719; Molecular chaperone (DnaJ superfamily) [Posttranslational modification, protein turnover, chaperones] (98.36/1.00e-06)<br><br>KOG3442; Uncharacterized conserved protein [Function unknown] (98.49/1.20e-06)<br><br>KOG0723; Molecular chaperone (DnaJ superfamily) [Posttranslational modification, protein turnover, chaperones] (98.49/1.40e-06)	DnaJ domain containing protein
103	88441	88223	-	hp	hp	-	-	KOG3735; Tropomodulin and leiomodulin [Cytoskeleton](80.21/4.40e+00)	hp
104	88672	88454	-	conserved phage protein [*Bacillus* phage Gamma]<br><br>putative membrane protein [*Bacillus* phage Bcp1]<br><br>putative membrane protein [*Bacillus* phage vB_BceM_Bc431v3]	2.0e-06<br><br>4.0e-05<br><br>7.0e-05	-	-	PF13239.7; 2TM; 2TM domain(86.7/6.30e+00)	hp
105	89054	88812	-	hp	hp	-	-	('-', '-')	hp
106	89420	89067	-	putative GTP binding protein [*Bacillus* phage PBC4]	1.0e-37<br><br>1.0e-36	DUF2493 (1-66)	1.50e-11	COG0758; Smf; Predicted Rossmann fold nucleotide-binding protein DprA/Smf involved in DNA uptake (99.83/1.40e-18)	putative nucleotide-binding protein

				putative GTP binding protein [*Bacillus* phage pW4]<br><br>putative ssDNA binding protein [*Bacillus* phage vB_BspM_MarvelLand]	8.0e-35			d2nx2a1; c.129.1.2 (A:2-177) Hypothetical protein YpsA {*Bacillus subtilis* [TaxId: 1423]} (99.82/2.40e-18)<br><br>COG4474; YoqJ; Uncharacterized SPBc2 prophage-derived protein YoqJ [Mobilome: prophages, transposons]. (99.77/9.50e-17)	
107	89836	89417	-	hp	hp	-	-	('-', '-')	hp
108	90045	89833	-	hp	hp	-	-	('-', '-')	hp
109	90600	90097	-	hp	hp	-	-	('-', '-')	hp
110	90965	90600	-	host recBCD nuclease inhibitor [*Enterococcus* phage EF-P29]<br><br>inihibitor of recBCD nuclease [*Escherichia* phage ECA2]<br><br>putative 5.9 protein [*Yersinia* phage phiYeO3-12]	1.0e-07<br><br>6.0e-07<br><br>6.0e-07	PHA00442 (83-121)	2.97e-07	('-', '-')	hp
111	91292	91017	-	-	-	-	-	('-', '-')	hp
112	91521	91312	-	-	-	-	-	PF00794.19; PI3K_rbd; PI3-kinase family, ras-binding domain (61.01/5.20e+00)	hp
113	91794	91522	-	hp	hp	-	-	cd00927; Cyt_c_Oxidase_VIc; Cytochrome c oxidase subunit VIc. Cytochrome c oxidase (CcO), the terminal oxidase in the respiratory (86.04/1.40e+00)	hp
114	92047	91787	-	hp	hp	-	-	PF15597.7; Imm59; Immunity protein 59(63.14/6.80e+00)	hp
115	92616	92059	-	putative guanylate kinase [*Bacillus* phage PBC2]	8.0e-72<br><br>2.0e-71	guanyl_kin (3-164)<br><br>GMPK	6.88e-43<br><br>3.03e-34	d1kjwa2; c.37.1.1 (A:526-724) Guanylate kinase-like domain of Psd-95 {Norway rat (Rattus norvegicus) [TaxId: 10116]} (99.88/1.30e-19)	guanylate kinase

				guanylate kinase [*Bacillus* phage Izhevsk]<br><br>putative guanylate kinase [*Bacillus* phage pW2]	4.0e-69	(3-120)<br><br>GuKc<br>(10-164)	8.77e-21	d1t0hb_; c.37.1.1 (B:) Guanylate kinase-like domain of the L-type calcium channel {Norway rat (Rattus norvegicus) [TaxId: 10116]} (99.86/1.60e-19)<br><br>COG0194; Gmk; Guanylate kinase [Nucleotide transport and metabolism]. (99.87/9.00e-19)	
116	92786	92616	-	hp	hp	-	-	('-', '-')	hp
117	93054	92788	-	putative signal peptide-containing lipoprotein [*Bacillus* phage Izhevsk]	7.0e-08	-	-	('-', '-')	hp
118	93315	93067	-	gp48 [*Bacillus* virus G]	9.0e-11	-	-	('-', '-')	hp
119	93851	93345	-	-	-	-	-	('-', '-')	hp
120	94070	93861	-	-	-	-	-	COG0776; HimA; Bacterial nucleoid DNA-binding protein [Replication, recombination and repair]. (95.74/3.90e-02)<br><br>d1p71a_; a.55.1.1 (A:) HU protein {*Anabaena* sp. [TaxId: 1167]} (95.58/4.90e-02)<br><br>d1mula_; a.55.1.1 (A:) HU protein {*Escherichia coli* [TaxId: 562]} (95.44/5.80e-02)	HU-like DNA-binding protein
121	94287	94081	-	-	-	-	-	('-', '-')	hp
122	95202	94387	-	trimeric dUTP diphosphatase [*Bacillus* phage Izhevsk]	1.0e-99<br><br>2.0e-51	Dut<br>(104-271)<br><br>trimeric_dUTPase<br>(124-203)	7.55e-16<br><br>2.15e-09	KOG3370; dUTPase [Nucleotide transport and metabolism] (99.94/4.40e-26)	dUTPase

				deoxyuridine 5'-triphosphate nucleotidohydrolase [*Bacillus* phage vB_BcoS-136]<br><br>putative DUTP diphosphatase Dut [*Brevibacillus* phage Sundance]	9.0e-42	Dut<br>(123-271)	8.62e-07	COG0756; Dut; dUTPase [Nucleotide transport and metabolism, Defense mechanisms]. (99.93/3.00e-24)<br><br>d1sixa1; b.85.4.1 (A:1-144) Deoxyuridine 5'-triphosphate nucleotidohydrolase (dUTPase) {*Mycobacterium tuberculosis*} (99.92/1.20e-23)	
123	95590	95279	-	glutaredoxin/thioredoxin [*Bacillus* phage BigBertha]<br><br>thioredoxin [*Bacillus* phage Troll]<br><br>thioredoxin [*Bacillus* phage Riley]	4.0e-07<br><br>5.0e-07<br><br>5.0e-07	TRX_family<br>(10-79)	4.96e-06	d1a8la1; c.47.1.2 (A:1-119) Protein disulfide isomerase, PDI {*Pyrococcus furiosus* [TaxId: 2261]} (99.62/3.00e-14)<br><br>d1dbya1; c.47.1.1 (A:2-107) Thioredoxin {Green alga (*Chlamydomonas reinhardtii*) [TaxId: 3055]} (99.62/4.00e-14)<br><br>d3diea1; c.47.1.1 (A:1-104) Thioredoxin {*Staphylococcus aureus* [TaxId: 1280]} (99.61/8.10e-14)	thioredoxin
124	96738	95674	-	ribonucleotide-diphosphate reductase [*Bacillus* phage PBC2]<br><br>putative ribonucleotide-diphosphate reductase [*Brevibacillus* phage Sundance]<br><br>gp83 [*Bacillus* virus G]	0.0e+00<br><br>4.0e-129<br><br>2.0e-123	RNR_1b_NrdF<br>(28-350)<br><br>Ribonuc_red_sm<br>(30-311)	4.75e-173<br><br>6.99e-91	KOG1567; Ribonucleotide reductase, beta subunit [Nucleotide transport and metabolism] (100.0/2.40e-47)<br><br>COG0208; NrdF; Ribonucleotide reductase beta subunit, ferritin-like domain [Nucleotide transport and metabolism]. (100.0/6.30e-45)<br><br>d3dhza_; a.25.1.2 (A:) Ribonucleotide reductase R2 {*Corynebacterium ammoniagenes* [TaxId: 1697]} (100.0/3.50e-43)	ribonucleoside-diphosphate reductase class 1b, beta subunit

125	98841	96754	-	ribonucleotide reductase [*Bacillus* phage PBC2]<br><br>NrdE-like class 1b ribonucleotide reductase, alpha subunit [*Bacillus* phage Izhevsk]<br><br>putative ribonucleoside-diphosphate reductase subunit alpha [*Brevibacillus* phage Sundance]	0.0e+00<br><br>0.0e+00<br><br>0.0e+00	PRK07632 (3-695)<br><br>Ribonuc_red_lgC (163-679)<br><br>RNR_I (113-682)	0e+00<br><br>0e+00<br><br>0e+00	KOG1112; Ribonucleotide reductase, alpha subunit [Nucleotide transport and metabolism] (100.0/3.80e-104)<br><br>d1peqa2; c.7.1.2 (A:175-699) R1 subunit of ribonucleotide reductase, C-terminal domain {*Salmonella* typhimurium [TaxId: 90371]} (100.0/1.50e-92)<br><br>COG0209; NrdA; Ribonucleotide reductase alpha subunit [Nucleotide transport and metabolism]. (100.0/1.00e-91)	ribonucleotide-diphosphate reductase class 1b, alpha subunit
126	99057	98860	-	hp	hp	-	-	d1ix2a_; b.1.18.17 (A:) Copper resistance protein C (CopC, PcoC) {*Escherichia coli* [TaxId: 562]} (75.26/4.70e+00)	hp
127	99436	99062	-	putative ribonucleotide reductase [*Bacillus* phage PBC2]<br><br>NrdI-like protein of class Ib ribonucleotide reductase operon [*Bacillus* phage Izhevsk]<br><br>gp81 [*Bacillus* virus G]	2.0e-67<br><br>2.0e-44<br><br>2.0e-38	nrdI (1-117)<br><br>Flavodoxin_NdrI (1-118)	9.93e-41<br><br>1.46e-32	COG1780; NrdI; Protein involved in ribonucleotide reduction [Nucleotide transport and metabolism]. (99.82/7.60e-18)<br><br>d1rlja1; c.23.5.7 (A:1-126) Flavoprotein NrdI {*Bacillus subtilis* [TaxId: 1423]} (99.74/7.90e-16)<br><br>d1ykga1; c.23.5.2 (A:63-208) Sulfite reductase alpha-component CysJ N-terminal domain {*Escherichia coli* [TaxId: 562]} (99.62/8.80e-14)	ribonucleoside-diphosphate reductase assembly flavoprotein NrdI
128	99963	99523	-	DUF1653 domain-containing protein [*Bacillus* phage Izhevsk]	7.0e-53	DUF1653 (1-106)	5.09e-09	COG4728; COG4728; Uncharacterized protein, DUF1653 family [Function unknown]. (99.83/1.90e-20)	DUF1653 domain-containing protein

			-	putative TonB box-like protein [*Brevibacillus* phage Sundance]	2.0e-08			PF07866.12; DUF1653; Protein of unknown function (DUF1653) (99.56/2.50e-14)	
129	100865	100038	-	putative HflC/HflK family inner membrane protein [*Brevibacillus* phage Sundance]<br><br>HflK protein [*Bacillus* phage vB_BcoS-136]<br><br>membrane protein [*Enterococcus* phage PEf771]	2.0e-79<br><br>9.0e-79<br><br>2.0e-50	SPFH_prohibitin (29-223)<br><br>Band_7 (29-208)	2.68e-38<br><br>1.16e-18	KOG3083; Prohibitin [Posttranslational modification, protein turnover, chaperones] (100.0/1.70e-32)<br><br>KOG3090; Prohibitin-like protein [Posttranslational modification, protein turnover, chaperones] (100.0/1.80e-32)<br><br>KOG2962; Prohibitin-related membrane protease subunits [General function prediction only] (100.0/3.50e-30)	prohibitin family protein
130	103240	101099	-	DNA topoisomerase [*Bacillus* phage PBC2]<br><br>DNA gyrase subunit A [*Bacillus* phage Izhevsk]<br><br>DNA topoisomerase [*Bacillus* phage pW2]	0.0e+00<br><br>0.0e+00<br><br>0.0e+00	GyrA (4-695)<br><br>PRK05561 (4-656)<br><br>gyrA (4-713)	4.51e-167<br><br>1.66e-166<br><br>1.65e-144	COG0188; GyrA; DNA gyrase/topoisomerase IV, subunit A [Replication, recombination and repair]. (100.0/4.30e-118)<br><br>d3k9fa_; e.11.1.1 (A:) DNA Gyrase A {*Pneumococcus* (*Streptococcus pneumoniae*) [TaxId: 1313]} (100.0/4.80e-100)<br>d4ckka_; e.11.1.1 (A:) DNA Gyrase A {*Escherichia coli* [TaxId: 562]} (100.0/1.60e-96)	DNA gyrase/topoisomerase IV, subunit A
131	105247	103286	-	DNA gyrase subunit B [*Bacillus* phage vB_BcoS-136]<br><br>putative DNA gyrase [*Geobacillus* virus E3]	0.0e+00<br><br>0.0e+00	GyrB (1-643)<br><br>TOP2c (33-644)	9.70e-161<br><br>2.96e-139<br><br>1.68e-50	COG0187; GyrB; DNA gyrase/topoisomerase IV, subunit B [Replication, recombination and repair]. (100.0/4.80e-105)	DNA gyrase/topoisomerase IV, subunit B

				DNA topoisomerase [*Bacillus* phage PBC2]	0.0e+00	TOPRIM_TopoIIA _GyrB (429-550)		KOG0355; DNA topoisomerase type II [Chromatin structure and dynamics] (100.0/6.30e-65)<br><br>d3k9fc_; e.78.1.1 (C:) DNA gyrase beta-prime domain {*Pneumococcus* (*Streptococcus pneumoniae*) [TaxId: 1313]} (100.0/2.60e-57)	
132	106068	105307	-	putative nucleotidyltransferase [*Bacillus* phage Izhevsk]<br><br>putative nucleotidyltransferase [*Bacillus* phage vB_BcoS-136]<br><br>putative nucleotidyltransferase [*Brevibacillus* phage Sundance]	9.0e-119<br><br>5.0e-77<br><br>1.0e-61	Nuc-transf (9-118)	2.19e-04	PF10127.10; RlaP; RNA repair pathway DNA polymerase beta family (99.98/4.40e-31)<br><br>COG3541; YcgL; Predicted nucleotidyltransferase [General function prediction only]. (99.97/5.60e-29)<br><br>d1knya2; d.218.1.1 (A:1-125) Kanamycin nucleotidyltransferase (KNTase), N-terminal domain {*Staphylococcus aureus* [TaxId: 1280]} (97.24/1.10e-03)	putative nucleotidyltransferase
133	106692	106198	-	hp	hp	-	-	COG1590; Tyw3; tRNA (Phe) wybutosine-synthesizing methylase Tyw3 [Translation, ribosomal structure and biogenesis]. (96.44/1.70e-03)<br><br>PF02676.15; TYW3; Methyltransferase TYW3(96.43/2.90e-03)<br><br>KOG1228; Uncharacterized conserved protein [Function unknown](96.45/3.30e-03)	hp
134	107300	106692	-	NADAR family protein [*Bacillus* phage pW2]	6.0e-80	DUF1768 (46-196)	8.25e-50	COG3236; ybiA; N-glycosylase of 5-amino-6-ribosylamino-2,4-	NADAR family protein

				GTP cyclohydrolase [*Serratia* phage BF]<br><br>Uncharacterized protein COG3236 [*Yersinia* phage fHe-Yen9-04]	4.0e-39<br><br>4.0e-39	ribofla_fusion (45-196)<br><br>ybiA (40-198)	1.91e-47<br><br>3.92e-36	pyrimidinedione 5-phosphate (riboflavin biosynthesis damage control) (100.0/1.50e-35)<br><br>d2b3wa1; d.336.1.1 (A:1-160) Hypothetical protein YbiA {*Escherichia coli* [TaxId: 562]} (100.0/2.90e-34)<br><br>PF08719.12; NADAR; NADAR domain (100.0/8.10e-32)	
135	107760	107305	-	hp	hp	-	-	('-', '-')	hp
136	108367	107753	-	hp	hp	-	-	('-', '-')	hp
137	108596	108393	-	-	-	-	-	('-', '-')	hp
138	108876	108628	-	hp	hp	-	-	('-', '-')	hp
139	109058	108873	-	uncharacterized conserved protein [*Staphylococcus* phage SpaA1]<br><br>gp243 [*Bacillus* phage W.Ph.]	8.0e-19<br><br>9.0e-11	-	-	('-', '-')	hp
140	109421	109062	-	hp	hp	-	-	('-', '-')	hp
141	109647	109408	-	-	-	-	-	cd01825; SGNH_hydrolase_peri1; SGNH_peri1; putative periplasmic member of the SGNH-family of hydrolases, a diverse family of lipa (74.83/2.50e+00)	hp
142	109865	109701	-	-	-	-	-	('-', '-')	hp
143	110129	109878	-	hp	hp	-	-	PF11360.9; DUF3110; Protein of unknown function (DUF3110)(92.1/4.50e-01)	hp
144	110306	110148	-	gp49 [*Bacillus* phage TP21-L]	6.0e-10	-	-	d1lv3a_; g.39.1.9 (A:) Hypothetical zinc finger protein YacG {*Escherichia coli* [TaxId: 562]} (74.76/3.60e-01)	Zn-ribbon domain containing protein

145	110499	110320	-	hp	hp	-	-	d1pava_; d.68.3.3 (A:) Hypothetical protein Ta1170/Ta1414 {*Thermoplasma acidophilum* [TaxId: 2303]} (96.46/2.00e-02)<br><br>COG0425; TusA; TusA-related sulfurtransferase [Posttranslational modification, protein turnover, chaperones]. (96.04/4.10e-02)<br><br>cd03422; YedF; YedF is a bacterial SirA-like protein of unknown function. SirA (also known as UvrY, and YhhP) belongs to a family (95.96/6.50e-02)	hp
146	110707	110528	-	-	-	-	-	('-', '-')	hp
147	111269	110733	-	hp	hp	-	-	('-', '-')	hp
148	111444	111271	-	-	-	-	-	PF00763.24; THF_DHG_CYH; Tetrahydrofolate dehydrogenase/cyclohydrolase, catalytic domain (76.55/3.20e+00)	hp
149	111607	111449	-	hp	hp	-	-	('-', '-')	hp
150	111783	111607	-	hp	hp	-	-	('-', '-')	hp
151	112351	111785	-	hp	hp	-	-	d1qp2a_; b.34.4.2 (A:) Photosystem I accessory protein E (PsaE) {Nostoc sp., strain pcc8009 [TaxId: 1180]} (99.96/6.30e-30)<br><br>d4kt0e_; b.34.4.2 (E:) Photosystem I accessory protein E (PsaE) {Synechocystis sp. PCC 6803 [TaxId: 1148]} (99.94/2.00e-27)	PsaE-like protein

								PF02427.18; PSI_PsaE; Photosystem I reaction centre subunit IV / PsaE(99.93/2.00e-26)	
152	112902	112348	-	excinuclease [*Bacillus* phage Izhevsk]	3.0e-89	GIY-YIG_UvrC_Cho (7-79)<br><br>UvrC (7-91)	2.92e-16<br><br>1.41e-11	COG0322; UvrC; Excinuclease UvrABC, nuclease subunit [Replication, recombination and repair]. (99.83/2.00e-19)<br><br>cd10435; GIY-YIG_RE_Eco29kI_like; Catalytic GIY-YIG domain of type II restriction endonucleases R.Eco29kI, R.Cfr42I, and similar (99.33/1.20e-11)<br><br>cd10453; GIY-YIG_RE_Cfr42I; Catalytic GIY-YIG domain of type II restriction enzyme R.Cfr42I and similar proteins.(99.32/1.40e-11)	GIY-YIG endonuclease
153	113269	112895	-	hp	hp	-	-	('-', '-')	hp
154	113576	113271	-	hp	hp	-	-	('-', '-')	hp
155	113930	113586	-	hp	hp	-	-	PF16339.6; DUF4969; Domain of unknown function (DUF4969) (97.74/9.30e-05)<br><br>COG5567; YifL; Predicted small periplasmic lipoprotein YifL (function unknown0 [Function unknown]. (97.48/1.80e-04)<br><br>COG4808; YehR; Uncharacterized lipoprotein YehR, DUF1307 family [Function unknown]. (97.51/4.30e-04)	signal peptide-containing lipoprotein
156	114289	113939	-	hp	hp	-	-	d2rb6a1; b.38.1.6 (A:25-76) Uncharacterized protein SO0963	signal peptide-containing lipoprotein

								{*Shewanella oneidensis* [TaxId: 70863]} (99.46/6.40e-14)<br><br>d2k57a1; b.38.1.6 (A:2-53) Uncharacterized protein PSPPH2109 {*Pseudomonas syringae* [TaxId: 317]} (99.45/9.10e-14)<br><br>d3bdua1; b.38.1.6 (A:2-54) Uncharacterized protein ECA1013 {*Erwinia carotovora* [TaxId: 554]} (99.46/1.20e-13)	
157	114536	114306	-	hp	hp	-	-	PF13179.7; DUF4006; Family of unknown function (DUF4006)(87.6/1.90e+00)	hp
158	115007	114549	-	macro domain protein [*Bacillus* phage vB_BcoS-136]<br><br>Poa1p-like Appr-1"-p processing protein [*Bacillus* phage Izhevsk]<br><br>Appr-1-p processing-like protein [*Geobacillus* virus E3]	5.0e-55<br><br>9.0e-54<br><br>9.0e-42	Macro_Poa1p-like (1-144)	1.54e-19	COG2110; YmdB; O-acetyl-ADP-ribose deacetylase (regulator of RNase III), contains Macro domain [Translation, ribosomal structure (99.74/4.40e-16)<br><br>cd03331; Macro_Poa1p_like_SNF2; Macro domain, Poa1p_like family, SNF2 subfamily. The macro domain is a high-affinity ADP-ribose b(99.75/1.40e-15)<br><br>d1yd9a1; c.50.1.2 (A:6-193) Histone macro-H2a1.1 {Norway rat (*Rattus norvegicus*) [TaxId: 10116]} (99.73/1.70e-15)	macro domain containing protein
159	115225	115040	-	hp	hp	-	-	PF10925.9; DUF2680; Protein of unknown function (DUF2680) (92.99/1.10e+00)	hp
160	115608	115237	-	hp	hp	-	-	PF03961.14; FapA; Flagellar Assembly Protein A(95.27/2.00e-01)	hp

161	116095	115610	-	hp	hp	-	-	('-', '-')	hp
162	116350	116126	-	hp	hp	-	-	PF11446.9; DUF2897; Protein of unknown function (DUF2897) (94.44/3.30e-01)	hp
163	117123	116347	-	SpoT-like bifunctional (p)ppGpp synthase/hydrolase [*Bacillus* phage Izhevsk]<br><br>GTP pyrophosphokinase [*Bacillus* phage vB_BcoS-136]<br><br>GTP pyrophosphokinase [*Bacillus* phage pW2]	3.0e-100<br><br>2.0e-90<br><br>8.0e-90	spoT_relA(38-167)<br><br>PRK11092(34-167)<br><br>SpoT(24-167)	3.95e-14<br><br>8.61e-14<br><br>5.62e-13	KOG1157; Predicted guanosine polyphosphate pyrophosphohydrolase/synthase [Signal transduction mechanisms] (100.0/3.30e-35)<br><br>COG0317; SpoT; (p)ppGpp synthase/hydrolase, HD superfamily [Signal transduction mechanisms, Transcription]. (100.0/9.00e-35)<br><br>d1vj7a1; a.211.1.1 (A:5-196) Stringent response-like protein RelA N-terminal domain {*Streptococcus equisimilis* [TaxId: 119602]} (99.97/8.80e-30)	SpoT-like bifunctional (p)ppGpp synthase/hydrolase
164	118916	117231	-	single-stranded-DNA-specific exonuclase RecJ [*Bacillus* phage PBC2]<br><br>RecJ-type single-stranded DNA-specific exonuclease [*Bacillus* phage Izhevsk]<br><br>putative SPBc2 prophage-derived single-strand DNA-specific exonuclease [*Bacillus* phage vB_BcoS-136]	0.0e+00<br><br>0.0e+00<br><br>0.0e+00	recJ (19-556)<br><br>RecJ (43-458)<br><br>PRK11070 (1-424)	1.04e-83<br><br>2.05e-63<br><br>3.35e-39	COG0608; RecJ; Single-stranded DNA-specific exonuclease, DHH superfamily, may be involved in archaeal DNA replication intiation [(100.0/4.70e-53)<br><br>d1ir6a_; c.107.1.2 (A:) Exonuclease RecJ {*Thermus thermophilus* [TaxId: 274]} (100.0/2.40e-40)<br><br>COG1107; COG1107; Archaea-specific RecJ-like exonuclease, contains DnaJ-type Zn finger domain [Replication, recombination and rep (100.0/4.70e-34)	RecJ-type single-stranded DNA-specific exonuclease
165	119579	119337	-	hp	hp	-	-	('-', '-')	hp

166	120158	119679	-	hp	hp	-	-	('-', '-')	hp
167	121349	120246	-	putative DNA primase [*Bacillus* phage PBC2]<br><br>DNA primase [*Bacillus* phage vB_BcoS-136]<br><br>DNA primase [*Bacillus* phage pW2]	0.0e+00<br><br>6.0e-175<br><br>9.0e-146	PRK08624 (9-298)<br><br>DnaG (153-297)<br><br>dnaG (83-297)	8.52e-22<br><br>4.04e-08<br><br>2.01e-07	COG0358; DnaG; DNA primase (bacterial type) [Replication, recombination and repair]. (100.0/1.90e-32)<br><br>d1dd9a_; e.13.1.1 (A:) DNA primase DnaG catalytic core {*Escherichia coli* [TaxId: 562]} (99.91/3.70e-22)<br><br>COG4643; COG4643; Uncharacterized domain associated with phage/plasmid primase [Mobilome: prophages, transposons]. (99.88/4.30e-21)	DNA primase
168	122892	121360	-	putative replicative DNA helicase [*Bacillus* phage PBC2]<br><br>replicative DNA helicase [*Bacillus* phage vB_BcoS-136]<br><br>DnaB-type replicative DNA helicase [*Bacillus* phage Izhevsk]	0.0e+00<br><br>0.0e+00<br><br>0.0e+00	DnaB (59-307)	1.84e-08	COG0305; DnaB; Replicative DNA helicase [Replication, recombination and repair]. (100.0/1.80e-36)<br><br>d1cr1a_; c.37.1.11 (A:) Gene 4 protein (g4p, DNA primase), helicase domain {Bacteriophage T7 [TaxId: 10760]} (99.92/5.80e-23)<br><br>PF03796.16; DnaB_C; DnaB-like helicase C terminal domain(99.9/6.50e-21)	DnaB-type replicative helicase
169	123513	122908	-	hp	hp	-	-	d1imla1; g.39.1.3 (A:1-28) Cysteine-rich (intestinal) protein, CRP, CRIP {Rat (Rattus rattus) [TaxId: 10117]} (92.91/7.90e-02)	hp
170	124143	123613	-	deoxynucleoside kinase [*Bacillus* phage PBC2]<br><br>deoxyribonucleoside kinase [*Bacillus* phage Izhevsk]	1.0e-87<br><br>4.0e-87	Tmk (1-136)<br><br>dNK (2-117)	4.30e-10<br><br>2.82e-09	d1tmka_; c.37.1.1 (A:) Thymidylate kinase {Baker's yeast (*Saccharomyces cerevisiae*) [TaxId: 4932]} (99.76/1.60e-16)	thymidylate kinase

				putative nucleotide kinase [*Bacillus* phage phi3Ts]	4.0e-32	AAA_18 (3-122)<br><br>DTMP_kinase (1-151)	5.45e-07<br><br>4.48e-05	cd01672; TMPK; Thymidine monophosphate kinase (TMPK), also known as thymidylate kinase, catalyzes the phosphorylation of thymidin(99.73/1.90e-15)<br><br>d4tmka_; c.37.1.1 (A:) Thymidylate kinase {*Escherichia coli* [TaxId: 562]} (99.69/1.20e-14)	
171	124899	124156	-	3D domain-containing protein [*Bacillus* phage Izhevsk]<br><br>cell wall-binding protein [*Bacillus* phage pW2]<br><br>cell-wall binding protein [*Bacillus* phage 0305phi8-36]	2.0e-139<br><br>4.0e-120<br><br>9.0e-39	3D_containing_proteins (150-247)<br><br>3D (189-247)<br><br>MAD (28-136)	1.62e-36<br><br>4.98e-18<br><br>9.04e-06	COG4942; EnvC; Septal ring factor EnvC, activator of murein hydrolases AmiA and AmiB [Cell cycle control, cell division, chromosom] (99.72/5.30e-16)<br><br>COG3883; CwlO1; Uncharacterized N-terminal domain of peptidoglycan hydrolase CwlO [Function unknown]. (99.04/4.10e-08)<br><br>COG0739; NlpD; Murein DD-endopeptidase MepM and murein hydrolase activator NlpD, contain LysM domain [Cell wall/membrane/envelope (97.74/9.80e-06)<br>PF06725.12; 3D; 3D domain (98.2/2.3e-05)	3D domain-containing protein
172	126085	124976	-	p-loop containing nucleoside triphosphate hydrolase [*Bacillus* phage PBC2]<br><br>nucleotide-binding protein [*Bacillus* phage Izhevsk]	0.0e+00<br><br>2.0e-162	AAA_24 (21-244)	1.82e-24	PF13479.7; AAA_24; AAA domain (99.8/2.20e-17)<br><br>d3ldaa2; c.37.1.11 (A:145-396) DNA repair protein Rad51, catalytic domain {Baker's yeast (*Saccharomyces cerevisiae*) [TaxId: 4932] (99.36/2.80e-11)	P-loop containing nucleoside triphosphate hydrolase

				p-loop containing nucleoside triphosphate hydrolase [*Bacillus* phage pW2]	1.0e-151			d1xp8a1; c.37.1.11 (A:15-282) RecA protein, ATPase-domain {*Deinococcus radiodurans* [TaxId: 1299]} (99.31/2.90e-11)	
173	127376	126234	-	putative single-stranded DNA binding protein [*Bacillus* phage PBC2]<br><br>single-stranded DNA-binding protein [*Bacillus* phage Izhevsk]<br><br>putative single-stranded DNA binding protein [*Bacillus* phage pW2]	5.0e-180<br><br>2.0e-111<br><br>1.0e-102	-	-	d1qvca_; b.40.4.3 (A:) ssDNA-binding protein {*Escherichia coli* [TaxId: 562]} (96.87/8.10e-02)	ssDNA-binding protein
174	127874	127497	-	hp	hp	-	-	('-', '-')	hp
175	128011	127889	-	-	-	-	-	('-', '-')	hp
176	128258	127992	-	hp	hp	-	-	d2icta_; a.35.1.3 (A:) Antitoxin HigA {*Escherichia coli* [TaxId: 562]} (98.98/1.60e-08)<br><br>COG5606; COG5606; Predicted DNA-binding protein, XRE-type HTH domain [General function prediction only]. (98.76/8.90e-08)<br><br>COG3655; YozG; DNA-binding transcriptional regulator, XRE family [Transcription]. (98.81/1.70e-07)	DNA-binding transcriptional regulator
177	129272	128274	-	DNA breaking-rejoining enzyme [*Bacillus* phage PBC2]	0.0e+00	INT_ICEBs1_C_like (151-215)	1.99e-04	COG4973; XerC; Site-specific recombinase XerC [Replication,	Xer family site-specific tyrosine recombinase

				DNA breaking-rejoining enzyme/site-specific recombinase [*Bacillus* phage pW2]<br><br>site-specific recombinase [*Bacillus* phage vB_BcoS-136]	2.0e-158<br><br>3.0e-133	XerC (32-245)	7.61e-04	recombination and repair]. (99.98/1.70e-29)<br><br>COG4974; XerD; Site-specific recombinase XerD [Replication, recombination and repair]. (99.97/2.00e-28)<br><br>COG4342; COG4342; Intergrase/Recombinase [Mobilome: prophages, transposons]. (99.89/1.40e-21)	
178	130099	129560	-	putative signal peptide-containing lipoprotein [*Bacillus* phage Izhevsk]	7.0e-25	-	-	PF08139.13; LPAM_1; Prokaryotic membrane lipoprotein lipid attachment site (97.25/4.70e-04)<br><br>PF16339.6; DUF4969; Domain of unknown function (DUF4969) (97.01/1.30e-03)<br><br>PF02402.17; Lysis_col; Lysis protein (96.93/1.60e-03)	signal peptide-containing lipoprotein
179	130713	130216	-	HepA-related protein [*Bacillus* phage vB_BcoS-136]	8.0e-59	-	-	PF07443.14; HARP; HepA-related protein (HARP)(97.4/6.00e-04)	hp
180	131154	130717	-	hp	hp	PHA02264(68-142)	3.59e-04	PF14076.7; DUF4258; Domain of unknown function (DUF4258) (99.28/5.60e-11)<br><br>COG2929; COG2929; Uncharacterized conserved protein, DUF497 family [Function unknown]. (98.31/1.10e-05)	hp

								PF04365.14; BrnT_toxin; Ribonuclease toxin, BrnT, of type II toxin-antitoxin system (97.46/3.30e-03)	
181	131561	131229	-	hp	hp	-	-	PF14076.7; DUF4258; Domain of unknown function (DUF4258) (99.17/3.20e-10)<br><br>COG2929; COG2929; Uncharacterized conserved protein, DUF497 family [Function unknown]. (98.18/4.20e-05)	hp
182	131935	131612	-	-	-	-	-	('-', '-')	hp
183	132430	131948	-	-	-	-	-	PF09413.11; DUF2007; Putative prokaryotic signal transducing protein (79.22/7.30e+00)	hp
184	132666	132430	-	hp	hp	-	-	('-', '-')	hp
185	132920	132663	-	-	-	-	-	('-', '-')	hp
186	133326	132934	-	-	-	-	-	('-', '-')	hp
187	133546	133328	-	hp	hp	-	-	PF02180.18; BH4; Bcl-2 homology region 4(84.51/1.70e+00)	hp
188	133808	133539	-	hp	hp	-	-	('-', '-')	hp
189	133993	133820	-	-	-	-	-	cd17067; RBD2_RGS12_like; Ras-binding domain (RBD) 2 of regulator of G protein signaling 12 (RGS12) and similar proteins. (69.87/7.40e+00)	hp
190	134363	133998	-	-	-	-	-	('-', '-')	hp
191	134604	134368	-	hp	hp	-	-	PF05952.13; ComX; *Bacillus* competence pheromone ComX(84.84/7.90e-01)	hp
192	134741	134631	-	-	-	-	-	COG1309; AcrR; DNA-binding transcriptional regulator, AcrR family [Transcription]. (68.22/2.70e+00)	hp
193	134965	134774	-	hp	hp	-	-	('-', '-')	hp

194	135182	134967	-	hp	hp	-	-	PF12119.9; DUF3581; Protein of unknown function (DUF3581) (65.33/6.30e+00)	hp
195	135307	135179	-	-	-	-	-	PF03811.14; Zn_Tnp_IS1; InsA N-terminal domain (99.07/8.10e-10)<br><br>COG3877; COG3877; Uncharacterized protein, DUF2089 family [Function unknown]. (98.52/3.10e-07)<br><br>PF13717.7; zinc_ribbon_4; zinc-ribbon domain (98.43/9.50e-07)	Zn-ribbon domain containing protein
196	135645	135307	-	hp	hp	YdjO(56-108)	1.07e-04	PF09629.11; YorP; YorP protein (97.86/5.60e-05)<br><br>d2l8da1; b.34.9.1 (A:5-66) Lamin-b receptor {Chicken (Gallus gallus) [TaxId: 9031]} (97.32/4.70e-03)<br><br>PF06442.12; DHFR_2; R67 dihydrofolate reductase (96.55/1.70e-02)	hp
197	136072	135647	-	gp248 [*Bacillus* virus G]<br><br>ORF 8 [*Haloarcula hispanica* virus SH1]<br><br>putative protein 3 [*Haloarcula hispanica icosahedral* virus 2]	7.0e-72<br><br>2.0e-14<br><br>2.0e-13	-	-	('-', '-')	hp
198	136244	136074	-	-	-	-	-	PF07563.12; DUF1541; Protein of unknown function (DUF1541) (64.02/2.70e+00)	hp

199	136443	136249	-	-	-	-	-	PF12660.8; zf-TFIIIC; Putative zinc-finger of transcription factor IIIC complex (72.71/2.50e+00)	hp
200	136787	136440	-	hp	hp	-	-	('-', '-')	hp
201	137956	136928	-	nicotinamide-nucleotide adenylyltransferase [*Bacillus* phage PBC2]<br><br>NadR type nicotinamide-nucleotide adenylyltransferase [*Bacillus* phage Izhevsk]<br><br>multifunctional transcriptional regulator/nicotinamide-nucleotide adenylyltransferase/ribosylnicotinamide kinase NadR [*Bacillus* phage pW2]	0.0e+00<br><br>0.0e+00<br><br>0.0e+00	PRK08099 (1-341)<br><br>AAA_28 (170-328)<br><br>NMNAT_NadR (5-164)	1.16e-79<br><br>1.45e-41<br><br>4.36e-23	d1lw7a1; c.26.1.3 (A:57-219) Transcriptional regulator NadR, NMN-adenylyltransferase domain {*Haemophilus influenzae* [TaxId: 727]} (99.83/4.90e-19)<br><br>cd02167; NMNAT_NadR; Nicotinamide/nicotinate mononucleotide adenylyltransferase of bifunctional NadR-like proteins. (99.76/1.50e-17)<br><br>COG0196; RibF; FAD synthase [Coenzyme transport and metabolism]. (99.67/5.70e-16)	nicotinamide-nucleotide adenylyltransferase/ribosylnicotinamide kinase NadR
202	138645	137956	-	nicotinamide mononucleotide transporter [*Bacillus* phage PBC2]<br><br>PnuC-like nicotinamide mononucleotide transporter [*Bacillus* phage Izhevsk]<br><br>nicotinamide mononucleotide transporter [*Bacillus* phage pW2]	5.0e-149<br><br>3.0e-124<br><br>5.0e-103	NMN_transporter (33-220)<br><br>PRK15397 (40-188)	8.36e-42<br><br>5.67e-14	COG3201; PnuC; Nicotinamide riboside transporter PnuC [Coenzyme transport and metabolism]. (100.0/3.80e-38)<br><br>PF04973.13; NMN_transporter; Nicotinamide mononucleotide transporter (100.0/1.90e-33)<br><br>PF04973.13; NMN_transporter; Nicotinamide mononucleotide transporter (98.54/5.20e-07)	PnuC-like nicotinamide mononucleotide transporter
203	138925	138764	-	hp	hp	-	-	('-', '-')	hp
204	139139	138909	-	hp	hp	-	-	('-', '-')	hp
205	139499	139317	-	hp	hp	-	-	PF18073.2; Rubredoxin_2; Rubredoxin metal binding domain (98.13/3.00e-06)	Zn-ribbon containing protein

								d1twfl_; g.41.9.2 (L:) RBP12 subunit of RNA polymerase II {Baker's yeast (*Saccharomyces cerevisiae*) [TaxId: 4932]} (98.21/3.40e-06)<br><br>cd00601; RNA_pol_P_RPB12; Eukaryotic RNA polymerases RPB12 subunit. RNA polymerases are multisubunit enzymes that synthesize RNA (98.09/8.60e-06)	
206	139741	139553	-	hp	hp	-	-	d1qy5a_; d.122.1.1 (A:) HSP90 {Dog (Canis familiaris) [TaxId: 9615]} (87.58/2.70e+00)	hp
207	139967	139743	-	hp	hp	-	-	('-', '-')	hp
208	140403	139972	-	putative methyltransferase type 11 [*Bacillus* phage PBC2]<br><br>putative methyltransferase type 11 [*Bacillus* phage B4]<br><br>methyltransferase [*Bacillus* phage Chotacabras]	7.0e-100<br><br>2.0e-81<br><br>5.0e-80	Methyltransf_25 (24-75)	5.36e-10	cd19250; AdoMet_MTase_I; class I SAM-binding methyltransferase. S-adenosylmethionine-dependent methyltransferases (SAM or AdoMet- (99.84/9.80e-20)<br><br>COG4627; COG4627; Predicted SAM-depedendent methyltransferase [General function prediction only]. (99.81/2.10e-18)<br><br>cd02487; AdoMet_MTase_I; class I SAM-binding methyltransferase. S-adenosylmethionine-dependent methyltransferases (SAM or AdoMet- (99.81/2.30e-18)	SAM-depedendent methyltransferase
209	140678	140418	-	hp	hp	-	-	PF09133.11; SANTA; SANTA (SANT Associated) (78.2/2.20e+00)	hp
210	140931	140659	-	-	-	-	-	d4pc3c1; a.5.2.2 (C:2-54) Elongation factor Ts (EF-Ts), N-terminal domain	putative translation elongation factor

								{*Escherichia coli* [TaxId: 562]} (97.04/1.20e-03)<br><br>cd14275; UBA_EF-Ts; UBA domain found in elongation factor Ts (EF-Ts) from bacteria, chloroplasts and mitochondria of eukaryotes. (96.92/2.10e-03)<br><br>d1aipc1; a.5.2.2 (C:2-53) Elongation factor Ts (EF-Ts), N-terminal domain {*Thermus thermophilus* [TaxId: 274]} (96.79/2.60e-03)	
211	141235	140987	-	hp	hp	-	-	COG1550; YlxP; Uncharacterized conserved protein YlxP, DUF503 family [Function unknown]. (72.88/6.00e+00)	hp
212	141431	141237	-	hp	hp	-	-	PF09868.10; DUF2095; Uncharacterized protein conserved in archaea (DUF2095) (88.72/6.60e-01)	hp
213	141595	141446	-	hp	hp	-	-	d1cuka1; a.5.1.1 (A:156-203) DNA helicase RuvA subunit, C-terminal domain {*Escherichia coli* [TaxId: 562]} (96.27/2.30e-02)<br><br>d1bvsa1; a.5.1.1 (A:148-203) DNA helicase RuvA subunit, C-terminal domain {*Mycobacterium leprae* [TaxId: 1769]} (96.27/3.30e-02)<br><br>d1ixsa_; a.5.1.1 (A:) DNA helicase RuvA subunit, C-terminal domain {*Thermus thermophilus* [TaxId: 274]} (96.21/4.10e-02)	RuvA C-terminal domain-containing protein

214	141716	141624	-	-	-	-	-	PF13965.7; SID-1_RNA_chan; dsRNA-gated channel SID-1(65.68/5.20e+00)	hp
215	142020	141745	-	hp	hp	-	-	COG4808; YehR; Uncharacterized lipoprotein YehR, DUF1307 family [Function unknown]. (97.33/4.40e-03)<br><br>PF08139.13; LPAM_1; Prokaryotic membrane lipoprotein lipid attachment site (94.38/6.50e-02)	signal peptide-containing lipoprotein
216	142426	142025	-	membrane-bound protein [*Bacillus* phage vB_BthM-Goe5]	9.0e-44	-	-	d1e0ta1; b.58.1.1 (A:70-167) Pyruvate kinase (PK) {*Escherichia coli* [TaxId: 562]} (88.45/1.70e+00)	hp
217	142626	142423	-	-	-	-	-	('-', '-')	hp
218	142882	142628	-	hp	hp	-	-	('-', '-')	hp
219	143117	142884	-	-	-	-	-	('-', '-')	hp
220	143502	143188	-	hp	hp	-	-	('-', '-')	hp
221	143674	143507	-	hp	hp	-	-	('-', '-')	hp
222	143883	143707	-	-	-	-	-	('-', '-')	hp
223	144197	143970	-	hp	hp	-	-	('-', '-')	hp
224	144439	144197	-	-	-	-	-	cd11629; HR1_FBP17; Protein kinase C-related kinase homology region 1 (HR1) Rho-binding domain of Formin Binding Protein 17. (91.86/2.00e+00)	hp
225	144599	144429	-	-	-	-	-	PF08509.12; Ad_cyc_g-alpha; Adenylate cyclase G-alpha binding domain (62.14/6.50e+00)	hp
226	144784	144611	-	hp	hp	-	-	('-', '-')	hp
227	144973	144812	-	-	-	-	-	PF09104.11; BRCA-2_OB3; BRCA2, oligonucleotide/oligosaccharide-binding, domain 3(88.6/2.10e+00)	hp
228	145306	144986	-	hp	hp	-	-	('-', '-')	hp

229	145726	145403	-	hp	hp	-	-	PF12824.8; MRP-L20; Mitochondrial ribosomal protein subunit L20(92.84/2.40e+00)	hp
230	146077	145790	-	hp	hp	-	-	d1e0fi_; g.3.15.2 (I:) Haemadin {Indian leech (*Haemadipsa sylvestris*) [TaxId: 13555]} (89.11/2.50e-01)	hp
231	146271	146077	-	hp	hp	-	-	('-', '-')	hp
232	146787	146356	-	-	-	-	-	d1vq8t1; b.34.5.1 (T:1-119) Ribosomal proteins L24 (L24p) {Haloarcula marismortui [TaxId: 2238]} (85.33/2.60e+00)	hp
233	146918	146808	-	-	-	-	-	PF05961.12; Chordopox_A13L; Chordopoxvirus A13L protein (92.83/3.20e-01)	hp
234	147067	146921	-	putative membrane protein [*Bacillus* phage vB_BceM_Bc431v3]	3.0e-23	-	-	PF16286.6; DUF4932; Domain of unknown function (DUF4932) (83.78/1.10e+00)	hp
235	147258	147064	-	-	-	-	-	PF13299.7; CPSF100_C; Cleavage and polyadenylation factor 2 C-terminal (80.2/4.00e+00)	hp
236	147464	147291	-	-	-	-	-	('-', '-')	hp
237	147633	147493	-	-	-	-	-	('-', '-')	hp
238	147863	147630	-	hp	hp	-	-	('-', '-')	hp
239	148360	147860	-	nucleotide pyrophosphohydrolase [*Bacillus* phage Anthony]<br><br>putative hydrolase [*Bacillus* phage Bastille]<br><br>putative hydrolase [*Bacillus* phage Eyuki]	3.0e-18<br><br>2.0e-17<br><br>5.0e-17	NTP-PPase_Af0060_like (83-141)	8.33e-10	d1vmga_; a.204.1.2 (A:) Hypothetical protein SSo12199 (SSo3215) {*Sulfolobus solfataricus* [TaxId: 2287]} (99.17/8.80e-10)<br><br>cd11542; NTP-PPase_u5; Nucleoside Triphosphate Pyrophosphohydrolase (EC 3.6.1.8) MazG-like domain found in a group of uncharacter (99.08/4.10e-09)	MazG nucleotide pyrophosphohydrolase domain containing protein

								cd11531; NTP-PPase_BsYpjD; Nucleoside Triphosphate Pyrophosphohydrolase (EC 3.6.1.8) MazG-like domain putative pyrophosphatase Yp (99.02/5.10e-09)	
240	148591	148391	-	-	-	-	-	('-', '-')	hp
241	148779	148621	-	-	-	-	-	('-', '-')	hp
242	149051	148785	-	-	-	-	-	('-', '-')	hp
243	149304	149065	-	gp183 [*Bacillus* phage W.Ph.]	5.0e-23	-	-	('-', '-')	hp
244	149489	149331	-	-	-	-	-	cd16347; VOC_like; uncharacterized subfamily of the vicinal oxygen chelate (VOC) family. (91.75/1.80e-01)	hp
245	149866	149570	-	hp	hp	-	-	cd08058; MPN_euk_mb; Mpr1p, Pad1p N-terminal (MPN) domains with catalytic isopeptidase activity (metal-binding). (77.04/1.90e+00)	hp
246	150113	149955	-	hp	hp	-	-	PF12273.9; RCR; Chitin synthesis regulation, resistance to Congo red (94.92/5.00e-02)	hp
247	150434	150114	-	hp	hp	-	-	COG2182; MalE; Maltose-binding periplasmic protein MalE [Carbohydrate transport and metabolism]. (98.25/2.40e-05)<br><br>COG1653; UgpB; ABC-type glycerol-3-phosphate transport system, periplasmic component [Carbohydrate transport and metabolism]. (97.99/1.10e-04)<br><br>COG4150; CysP; ABC-type sulfate transport system, periplasmic component [Inorganic ion transport and metabolism]. (97.94/1.40e-04)	signal peptide-containing lipoprotein

248	150912	150436	-	-	-	-	-	('-', '-')	hp
249	151352	150990	-	hp	hp	-	-	('-', '-')	hp
250	151617	151354	-	hp	hp	-	-	PF18472.2; HP1451_C; HP1451 C-terminal domain (90.56/9.80e-01)	hp
251	151883	151641	-	-	-	-	-	d2gu3a2; d.17.1.6 (A:34-98) Uncharacterized protein YpmB {*Bacillus subtilis* [TaxId: 1423]} (83.11/5.70e+00)	hp
252	152285	151947	-	hp	hp	-	-	PF11774.9; Lsr2; Lsr2(81.79/1.40e+00)	hp
253	152596	152303	-	-	-	-	-	PF19150.1; DUF5832; Family of unknown function (DUF5832) (74.67/5.20e+00)	hp
254	153077	152679	-	putative peptidase [*Bacillus* phage Izhevsk]	6.0e-69	-	-	PF05379.12; Peptidase_C23; Carlavirus endopeptidase (96.71/1.30e-02)<br><br>PF05413.12; Peptidase_C34; Putative closterovirus papain-like endopeptidase (96.7/1.50e-02)	putative peptidase
255	153534	153091	-	hp	hp	-	-	PF14069.7; SpoVIF; Stage VI sporulation protein F(79.62/7.20e+00)	hp
256	153676	153551	-	-	-	-	-	d1je3a1; d.68.3.3 (A:21-97) hypothetical protein YedF (EC005) {*Escherichia coli* [TaxId: 562]} (80.14/1.80e+00)	hp
257	153928	153740	-	hp	hp	-	-	('-', '-')	hp
258	154168	154016	-	hp	hp	-	-	PF13121.7; DUF3976; Domain of unknown function (DUF3976) (80.95/4.20e+00)	hp
259	155640	154270	-	serine/threonine protein kinase [*Bacillus* phage Izhevsk]	2.0e-21	SPS1<br>(46-178)	1.77e-08	cd05039; PTKc_Csk_like; Catalytic domain of C-terminal Src kinase-like Protein Tyrosine Kinases. PTKs catalyze the transfer of th(99.92/9.60e-24)<br><br>cd05148; PTKc_Srm_Brk; Catalytic domain of the Protein Tyrosine Kinases,	serine/threonine protein kinase

								Src-related kinase lacking C-terminal regulatory tyrosin (99.92/1.80e-23)<br><br>cd14158; STKc_IRAK4; Catalytic domain of the Serine/Threonine kinase, Interleukin-1 Receptor Associated Kinase 4. (99.91/3.00e-23)	
260	156477	156085	-	hp	hp	-	-	('-', '-')	hp
261	156809	156531	-	hp	hp	-	-	('-', '-')	hp
262	158429	158653	+	-	-	-	-	('-', '-')	hp
263	158730	158972	+	hp	hp	-	-	('-', '-')	hp
264	159056	159493	+	hp	hp	-	-	('-', '-')	hp
265	159508	159624	+	hp	hp	-	-	('-', '-')	hp
266	159670	160044	+	hp	hp	-	-	('-', '-')	hp
267	160075	160656	+	hp	hp	-	-	('-', '-')	hp
268	160752	161003	+	gp175 [*Bacillus* phage W.Ph.]	2.0e-19	-	-	PF02426.17; MIase; Muconolactone delta-isomerase (89.11/2.40e+00)	hp
269	161075	161344	+	hp	hp	-	-	('-', '-')	hp
270	161378	161545	+	-	-	-	-	cd19818; Bbox1_ZBBX; B-box-type 1 zinc finger found in zinc finger B-box domain-containing protein 1 (ZBBX) and similar proteins. (78.02/3.00e-01)	hp
271	161570	161767	+	hp	hp	-	-	PF07862.12; Nif11; Nif11 domain (78.25/5.80e+00)	hp
272	162292	162693	+	-	-	-	-	PF16270.6; DUF4923; Lipocalin-like domain (DUF4923) (87.2/3.20e+00)	hp
273	162693	162851	+	hp	hp	-	-	PF16061.6; DUF4803; Domain of unknown function (DUF4803) (80.23/1.10e+00)	hp
274	162934	164007	+	tail tape measure protein [*Bacillus* phage PBC4]	4.0e-08	-	-	PF15843.6; DUF4719; Domain of unknown function (DUF4719) (69.65/6.70e+00)	hp

				tail tape measure protein [*Bacillus* phage pW4]<br><br>ubiquitin carboxyl-terminal hydrolase [Mimivirus LCMiAC02]	4.0e-06<br><br>7.0e-06				
275	164134	164904	+	F-box and regulator of chromosome condensation repeat protein [Pithovirus LCPAC102]<br><br>unknown similar to AMEV033 [Mythimna separata entomopoxvirus 'L']	2.0e-03<br><br>4.0e-03	-	-	('-', '-')	hp

Table S4. tRNA genes of *Bacillus* phage *Kirovirus kirovense* Kirov.

ORF№	Start codon	Stop codon	Strand	Product	Anticodon
1	19516	19590	+	tRNA-Asn	gtt
2	19596	19667	+	tRNA-Asp	gtc
3	19758	19828	+	tRNA-Gly	gcc
4	19945	20023	+	tRNA-Gln	ttg
5	20078	20148	+	tRNA-His	gtg

Table S5. *Kirovirus kirovense* Kirov and the most closely related phages.

№	Name	Genome Accession number	Genome length	GC-content, %	CDSs	Number of tRNAs/tmRNAs	BLASTn nucleotide identity to Kirov, %*	Proteins shared with Kirov**	
								number	%
1	*Bacillus* phage Kirov	NC_071041	165,383	35.5	275	5/0	-	-	
2	Bacillus phage PBC2	NC_071040	168,689	34.4	259	17/0	60.3	154	57.68
3	*Bacillus* phage Izhevsk	NC_071045	168,638	34.3	259	18/0	45.2	138	51.69
4	*Bacillus* phage Diildio	-	171,148	34.3	267	18/0	47.6	138	50.92
5	*Bacillus* phage vB_BanS-Tsamsa	NC_023007	168,876	34.3	267	19/0	45.8	137	50.55
6	*Bacillus* phage vB_BanS_MrDarsey	NC_071047	164,998	34.2	245	17/0	42.4	134	51.54
7	*Bacillus* phage vB_BanS_Chewbecca	NC_071048	161,151	34.3	237	20/0	41.5	130	50.78
8	*Bacillus* phage vB_BanS_Nate	NC_071044	166,879	33.6	264	16/0	39.2	131	48.60
9	*Bacillus* phage vB_BanS_Sophrita	NC_071042	167,995	32.4	259	4/0	37.2	118	44.20
10	*Bacillus* phage pW2	NC_071043	160,627	32.5	254	4/0	36.5	120	45.40
11	*Bacillus* phage vB_BcoS-136	NC_071049	160,590	32.2	241	18/0	26.9	97	37.60
12	*Bacillus* phage vB_BsuS_PJN02	NC_071050	165,868	33.6	230	13/0	23.9	84	33.27
13	*Geobacillus* virus E3	NC_029073	141,298	29.6	224	0/0	13.3	32	12.83
14	*Bacillus* phage TsarBomba	NC_028890	162,486	40.1	248	19/0	7.9	15	5.74
15	*Bacillus* phage BCU4	NC_047735	154,371	39.9	225	19/0	7.6	14	5.60
16	*Bacillus* phage BCPST	NC_070845	81,528	33.9	132	2/0	6.6	12	5.90
17	*Bacillus* phage PBC4	NC_070843	80,647	34.0	126	2/0	6.6	12	6.00
18	*Bacillus* phage pW4	NC_070844	80,919	34.0	133	2/0	6.6	12	5.88
19	*Bacillus* phage Basilisk	NC_070841	82,008	33.9	123	3/0	6.6	13	6.53
20	*Bacillus* phage v_B-Bak10	NC_070842	82,931	33.8	127	2/0	6.6	13	6.47
21	*Bacillus* phage Slash	NC_022774	80,382	35.2	111	0/0	0	6	3.11
22	*Bacillus* phage Staley	NC_022767	81,656	35.4	113	0/0	0	6	3.10

23	*Bacillus* phage Stahl	NC_028856	80,148	35.3	108	0/0	0	6	3.13
24	*Bacillus* phage Stills	NC_028777	80,798	35.5	107	0/0	0	6	3.14
25	*Bacillus* phage Troll	NC_022088	163,019	37.8	289	0/0	0	14	4.96
26	*Bacillus* phage BigBertha	NC_022769	165,238	37.8	288	0/0	0	13	4.62
27	*Bacillus* phage Bastille	NC_018856	153,962	38.1	271	7/0	0	11	4.03
28	*Bacillus* phage Evoli	NC_024207	159,656	38.1	287	7/0	0	11	3.92
29	*Bacillus* phage Spock	NC_022763	164,297	37.6	281	0/0	0	14	5.04
30	*Bacillus* phage B4	NC_018863	162,596	37.7	285	0/0	0	13	4.64

*Determined using BLASTn (multiplying % coverage by % identity); **Determined using GET_HOMOLOGUES (COGtriangles algorithm, -G -t 0 -C 75).

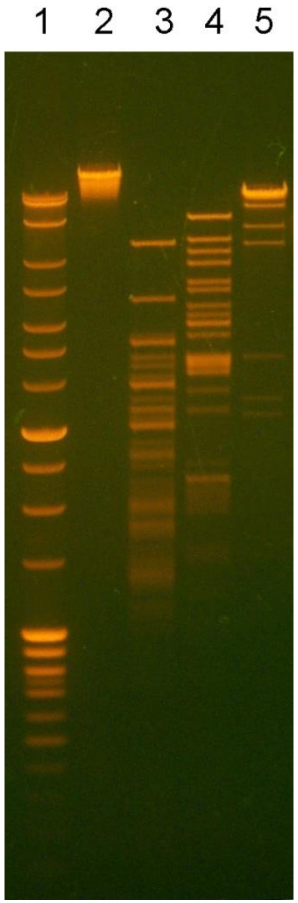

Figure S2. Restriction analysis of the phage genomic DNA. This is the original gel image used to generate Figure 2, A in the main text. Kodak EDAS 290 Gel Documentation System ("Kodak") was used to capture the image. 1 – molecular weight markers; 2 – intact phage DNA, 3 – HindIII, 4 – XbaI, 5 – PacI.

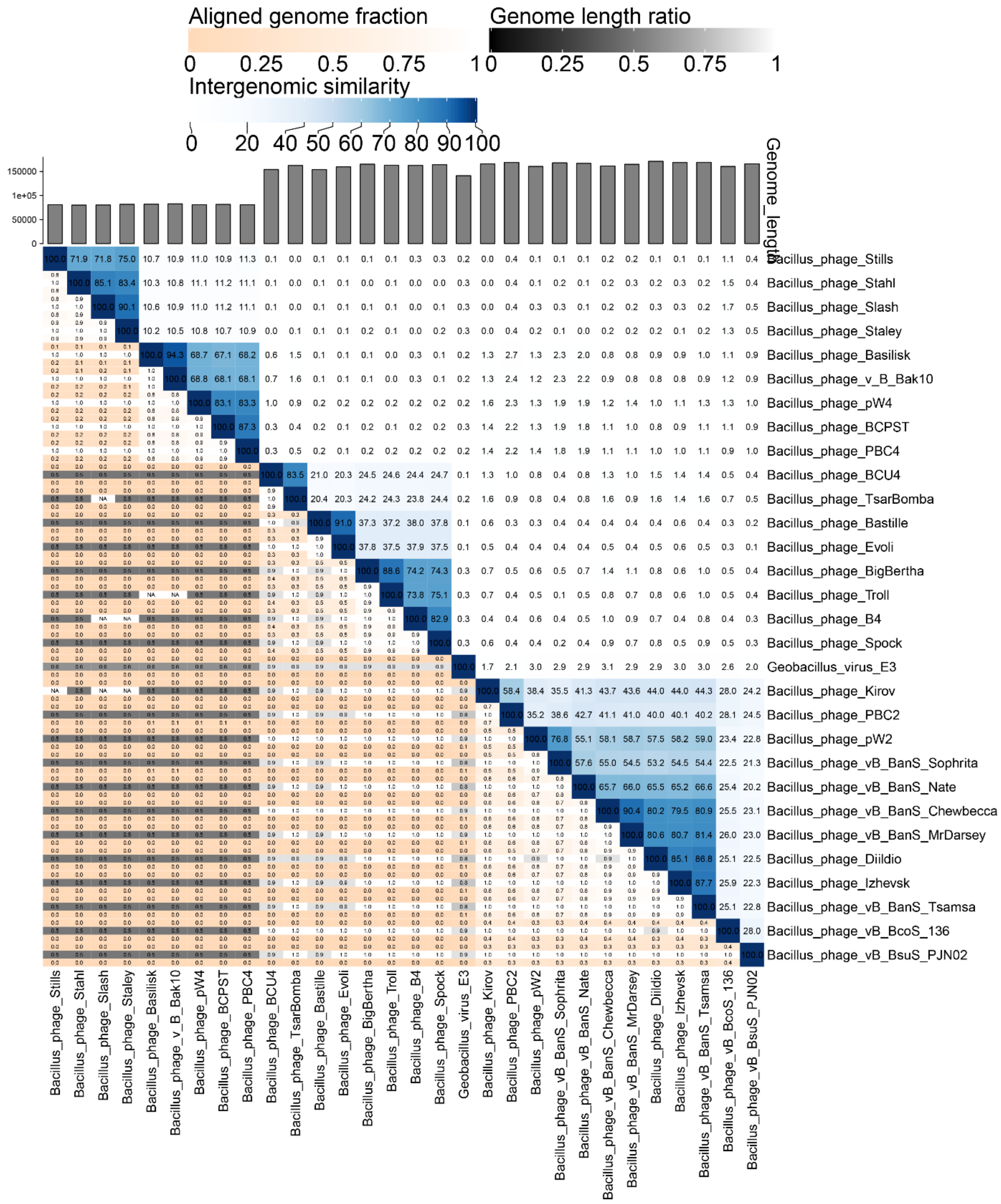

Figure S3. Heat map of 30 phage complete genome sequences generated by VIRIDIC. The right side of the figure displays intergenomic similarity between the 30 genomes using a blue gradient, where darker colors indicate a higher percentage of intergenomic similarity. In the left half, three different values are represented: the aligned genome fraction for the genome found in the row (top), genome length ratio (middle), and the aligned genome fraction for the genome found in the column (bottom). These values are ranked from 0 to 1, with 1 displayed in white.

Table S6. Reagent list.

No.	Reagent	Company name, address (city, country)/ other information
1	Acetic Acid	Kupavnareaktiv, Staraya Kupavna, Russia / chemically pure
2	Agar	Dia-M, Moscow, Russia / bacteriology grade, Cat.No. 3346.0500
3	Agarose	Bio-Rad, Richmond, CA, USA / standard electroendosmosis
4	Gelatin	Difco laboratories, Detroit, MI, USA /
5	Glycine	SERVA, Heidelberg, Germany / analytical grade
6	Calcium Chloride	Dalchempharm, Khabarovsk, Russia / pharm. grade
7	Cesium Chloride	Reachim, Staraya Kupavna, Russia / high purity grade
8	Magnesium Chloride	Aldosa, Moscow, Russia / chemically pure
9	PEG 8000	PanReac AppliChem, Darmstadt, Germany / pharm. grade
10	Hydrochloric Acid	Kupavnareaktiv, Staraya Kupavna, Russia / chemically pure
11	Magnesium Sulphate	Labochem international, Einhausen, Germany / pharm. grade
12	Sodium Acetate	PanReac, Darmstadt Germany / pharm. grade
13	Sodium Chloride	Kupavnareaktiv, Staraya Kupavna, Russia / chemically pure
14	Sodium Hydroxide	PanReac AppliChem, Darmstadt, Germany / pharm. grade
15	Sodium Phosphate, dibasic	PanReac, Darmstadt, Germany / pharm. grade
16	Sodium Phosphate, monobasic,	PanReac, Darmstadt, Germany / pharm. grade
17	Resazurin dye	Dia-M, Moscow, Russia / Cat.No. 3606.0025
18	Tris(hydroxymethyl)aminomethane	Sigma, Burlington, Massachusetts, United States / BioXtra
19	Trypton	Dia-M, Moscow, Russia / Cat.No. 3313.0500
20	Yeast Extract	Dia-M, Moscow, Russia / Cat.No. 3017.0500
